# Intraoperative respiratory and hemodynamic strategies for reducing nausea, vomiting, and pain after surgery: Systematic review and meta‐analysis

**DOI:** 10.1111/aas.14127

**Published:** 2022-08-22

**Authors:** Johanne M. Holst, Maibritt P. Klitholm, Jeppe Henriksen, Mikael F. Vallentin, Marie K. Jessen, Maria Bolther, Mathias J. Holmberg, Maria Høybye, Peter Carøe Lind, Asger Granfeldt, Lars W. Andersen

**Affiliations:** ^1^ Department of Anesthesiology and Intensive Care Aarhus University Hospital Aarhus Denmark; ^2^ Department of Clinical Medicine Aarhus University Aarhus Denmark; ^3^ Prehospital Emergency Medical Services Central Denmark Region Aarhus Denmark; ^4^ Research Center for Emergency Medicine Aarhus University Hospital Aarhus Denmark; ^5^ Department of Anesthesiology and Intensive Care Randers Regional Hospital Randers Denmark

**Keywords:** anesthesia, hemodynamic, respiratory, review, pain, nausea, vomiting, PONV

## Abstract

**Background:**

Despite improved medical treatment strategies, postoperative pain, nausea, and vomiting remain major challenges. This systematic review investigated the relationship between perioperative respiratory and hemodynamic interventions and postoperative pain, nausea, and vomiting.

**Methods:**

PubMed and Embase were searched on March 8, 2021 for randomized clinical trials investigating the effect of perioperative respiratory or hemodynamic interventions in adults undergoing non‐cardiac surgery. Investigators reviewed trials for relevance, extracted data, and assessed risk of bias. Meta‐analyses were performed when feasible. GRADE was used to assess the certainty of the evidence.

**Results:**

This review included 65 original trials; of these 48% had pain, nausea, and/or vomiting as the primary focus. No reduction of postoperative pain was found in meta‐analyses when comparing recruitment maneuvers with no recruitment, high (80%) to low (30%) fraction of oxygen, low (5–7 ml/kg) to high (9–12 ml/kg) tidal volume, or goal‐directed hemodynamic therapy to standard care. In the meta‐analysis comparing recruitment maneuvers with no recruitment maneuvers, patients undergoing laparoscopic gynecological surgery had less shoulder pain 24 h postoperatively (mean difference in the numeric rating scale from 0 to 10: −1.1, 95% CI: −1.7, −0.5). In meta‐analyses, comparing high to low fraction of inspired oxygen and goal‐directed hemodynamic therapy to standard care in patients undergoing abdominal surgery, the risk of postoperative nausea and vomiting was reduced (odds ratio: 0.45, 95% CI: 0.24, 0.87 and 0.48, 95% CI: 0.27, 0.85). The certainty in the evidence was mostly very low to low. The results should be considered exploratory given the lack of prespecified hypotheses and corresponding risk of Type 1 errors.

**Conclusion:**

There is limited evidence regarding the impact of intraoperative respiratory and hemodynamic interventions on postoperative pain or nausea and vomiting. More definitive trials are needed to guide clinical care within this area.

**Editorial Comment**

This systematic review assessed published clinical trials, which provide results concerning controlled intraoperative treatments for ventilation and circulatory management, and nausea/vomiting and pain outcomes after surgery. The study design was explorative—it did not include prospective hypotheses for specific treatments and outcomes, but rather general categories of interventions and outcomes. The authors found limited evidence, but some which might suggest that there could be a relation between inspired oxygen levels as well as goal‐directed circulatory therapy and postoperative nausea or vomiting.

## 1 INTRODUCTION

Severe postoperative pain, nausea, and vomiting remain major challenges. Despite improvements in treatment strategies, patients often experience these symptoms following surgery. Postoperative pain is associated with serious complications including increased incidence of pulmonary and cardiac complications, development of chronic pain, prolonged hospitalization, and increased mortality.^1^ Postoperative nausea and vomiting (PONV) is associated with prolonged time to hospital discharge.^2^

Pre‐, intra‐, and postoperatively, various medical interventions are used to minimize pain, nausea, and vomiting in the postoperative setting and avoid severe complications.^3^ Recent reviews have shown limited effects of specific intra‐operative respiratory and hemodynamic interventions on “hard” endpoints such as hospital length of stay and mortality.^4^, ^5^, ^6^, ^7^ However, identification of intraoperative respiratory and hemodynamic interventions and specific targets that can influence the development and intensity of postoperative pain, nausea, and vomiting would be valuable additions in a multimodal approach.

The aim of this hypothesis‐generating systematic review was to describe the literature regarding perioperative respiratory and hemodynamic interventions potentially affecting postoperative development of pain, nausea, and vomiting and explore any associations between these interventions and outcomes in meta‐analyses when possible.

## 2 METHODS

### 2.1 Protocol and registration

This paper is part of a larger systematic review project including clinical trials assessing various respiratory and hemodynamic interventions and targets for patients undergoing non‐cardiac surgery under general anesthesia. Previous manuscripts from this review project have focused on the outcomes mortality, length of stay, and postoperative complications.^4^, ^5^, ^6^, ^7^ In this manuscript, we focus on the outcomes of postoperative pain, nausea, and vomiting.

The protocol was uploaded to Figshare.com on June 11, 2020 and updated on August 19, 2020. The protocol is provided in the Supplementary Content. Reporting of this review followed the Preferred Reporting Items for Systematic Reviews and Meta‐Analyses (PRISMA) guidelines.^8^ The PRISMA checklist is provided in the Supplementary Content.

### 2.2 Eligibility criteria and population identification

The population of interest was adult patients under general anesthesia and invasive mechanical ventilation undergoing non‐cardiac surgery. Trials including caesarean sections, interventional radiology, very short duration of anesthesia (e.g., for electroconvulsive therapy), and surgery requiring one‐lung ventilation were excluded. Only English language publications were included, and there was no limitation regarding year of publication.

All specific intraoperative respiratory and hemodynamic interventions and targets (e.g., fraction of inspired oxygen, end‐tidal or arterial carbon dioxide level, tidal volume, positive end‐expiratory pressure, recruitment maneuver, goal‐directed hemodynamic therapy [GDHT], blood pressure, and different ventilation modes) were included. The comparator could be a different target or standard of care. Interventions occurring only pre‐ or postoperatively were not included. This manuscript includes all trials from the original search reporting the following outcomes: (1) postoperative pain, (2) administration of analgesics, and/or (3) nausea and vomiting.

### 2.3 Search strategy

PubMed and Embase were searched on July 24, 2020, and again on March 8, 2021. The full search strategies for both databases are provided in the protocol. The search included a combination of various text and indexing search terms for general anesthesia or surgery and the various respiratory and hemodynamic targets. The Cochrane sensitivity‐maximizing search strategy was used to identify randomized trials.^9^ The bibliographies of included articles were reviewed for additional relevant articles.

### 2.4 Study selection and data collection

Reviewers in pairs independently screened titles and abstracts retrieved from the systematic searches. Subsequently, articles were assessed in full text. Using a predefined standardized form, reviewers extracted data from the individual manuscripts. During all steps, any disagreement regarding eligibility and the extracted data was resolved via discussion between the reviewers and a third investigator if needed.

### 2.5 Outcome definitions and timeframes

Nausea, vomiting, PONV, and pain were the main outcomes in this manuscript. These outcomes were not prespecified in the protocol. *Nausea* is a subjectively unpleasant sensation associated with the awareness of the urge to vomit, whereas *vomiting* is an objective symptom; both are in general reported as incidences (i.e., yes/no) within a given timeframe. In some studies, nausea is quantified using a numeric rating scale (NRS), visual analogue scale (VAS), or categorized (e.g., none/mild/severe). VAS is an analog scale frequently presented as a 10 cm long line. The patients are asked to make a mark on the line corresponding to a particular symptom. NRS is a discrete numerical scale from 0 to 10 requiring that the patient choose a specific number to rate their symptoms. *PONV* is a composite outcome including nausea or vomiting.

*Pain* is a subjective symptom and is mostly reported using NRS or VAS (ranging from 0 to 10 or 0 to 100), where 0 is no pain and 10/100 is the worst pain imaginable. We report pain on the 0 to 10 scale. We also collected data on postoperative analgesic use. However, this data were very inconsistently reported and was therefore not considered further for meta‐analyses.

Various time points for the outcomes were reported in the included publications. Further details are provided in the Supplementary Content.

### 2.6 Risk of bias in individual trials

Using version 2 of the Cochrane risk‐of‐bias tool for randomized trials, risk of bias in the individual trials was independently assessed by two reviewers.^10^ Disagreements were resolved via discussion. Risk of bias was assessed for each outcome within a trial but is reported at the trial level as the highest risk of bias score across all outcomes. However, in most included trials, the risk of bias was the same across all outcomes. If the bias was different for the various outcomes, this was noted. Additional considerations about bias assessment are provided in the Supplementary Content.

### 2.7 Statistical analyses

Trials were assessed for clinical (i.e., participants, interventions, comparators, and outcomes) and methodological (i.e., study design or risk of bias) heterogeneity. If major heterogeneity was identified, no meta‐analyses were performed, and a descriptive summary of the trials was provided. A minimum of three trials reporting relevant events was required to perform meta‐analyses if there was no major heterogeneity. Meta‐analyses were performed using Review Manager 5.4.1 (Cochrane Collaboration, Nordic Cochrane Centre, Copenhagen, Denmark). Given that these meta‐analyses were not clearly specified in the protocol, they should be considered exploratory. For binary outcomes, we conducted meta‐analyses using Mantel–Haenszel random effects models. Results from these analyses are reported as odds ratios (ORs) with 95% confidence intervals (CI) with values below 1 indicating better outcomes in the intervention group. DerSimonian and Laird random effects meta‐analyses were used for continuous outcomes. Results from these analyses are presented as mean differences with 95% CI with values below zero indicating better outcomes in the intervention group. To allow for meta‐analyses, any continuous outcome reported as a median with a measure of variance (e.g., quartiles, range) was transformed to a mean and a standard deviation using the method described by Shi et al.^11^ Statistical heterogeneity was assessed using forest plots and I‐squared statistics.^12^ Based on the available data, exploratory subgroup analyses according to surgical characteristics were conducted.

### 2.8 Cumulative evidence

The certainty of the overall evidence for a given comparison and outcome was assessed using the Grading of Recommendations Assessment, Development and Evaluation (GRADE) methodology and classified within one of four categories: very low, low, moderate, or high certainty of evidence.^13^ Additional details are provided in the Supplementary Content. GRADE evaluation was only performed when a meta‐analysis was feasible. GRADEpro (McMaster University, 2020) was used for drafting of the GRADE tables.

## 3 RESULTS

Overall, the search identified 23,454 unique records of which 535 full‐text articles were assessed for eligibility. Of 209 manuscripts included in previous reviews,^4^, ^5^, ^6^, ^7^ 63 manuscripts including relevant outcomes for the current manuscript were identified. Review of references and previous reviews resulted in identification of two additional manuscripts, yielding a total of 65 manuscripts (Figure S1). An overview of the trials is provided in Table S1. Pain and/or nausea and vomiting were the primary focus in 31 of the manuscripts (48%). There was a large degree of heterogeneity among the included trials, for example in the type of surgery and the definition of the reported outcomes.

### 3.1 Fraction of inspired oxygen

Eighteen publications investigated a higher vs. a lower fraction of oxygen.^14^, ^15^, ^16^, ^17^, ^18^, ^19^, ^20^, ^21^, ^22^, ^23^, ^24^, ^25^, ^26^, ^27^, ^28^, ^29^, ^30^, ^31^ In the majority of the trials, a FiO_2_ of 80% was compared with a FiO_2_ of 30%.^14^, ^16^, ^17^, ^18^, ^19^, ^20^, ^21^, ^23^, ^24^, ^26^, ^27^, ^29^, ^30^, ^31^ A few trials investigated different mixtures of the combination of oxygen and nitrous oxide^19^, ^21^, ^25^ of which two were eligible for meta‐analyses.^19^, ^21^ Five trials contained more than 500 patients including two trials of 2012 and 4702 patients.^17^, ^18^, ^19^, ^20^, ^31^ The majority of the trials included surgery within the abdominal region (i.e., gastrointestinal, gynecologic, and urologic surgery). Additional information is reported in Tables S2–S4. All trials were assessed as having an intermediate risk of bias (Table S5).

**FIGURE 1:**
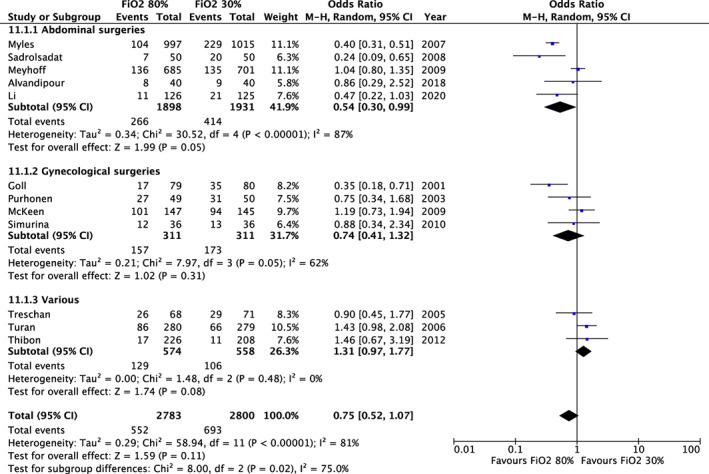
Fraction of inspired oxygen and postoperative nausea and vomiting. Results from random‐effects meta‐analyses of trials assessing fraction of inspired oxygen. Results are displayed as odds ratios (dots) with 95% confidence intervals (error bars). Values below 1 indicate reduced postoperative nausea and vomiting with 80% inspired oxygen. Subgroup analysis by abdominal surgeries, gynecological surgeries, and various surgeries.

In a meta‐analysis including 12 trials and 5583 patients, there was no clear difference in PONV (OR: 0.75; 95% CI: 0.52, 1.07; Figure 1) between a high and a low fraction of inspired oxygen. In the subgroup analysis according to surgery type (Figure 1), a FiO_2_ of 80% resulted in a decrease in PONV in trials primarily including patients having abdominal surgery (OR: 0.45, 95% CI: 0.24, 0.87), whereas there was no significant difference in PONV for laparoscopic gynecological surgery patients (OR 0.74, 95% CI: 0.41, 1.32), or other surgeries (OR: 1.31, 95%CI: 0.97, 1.77). The *p* value for a subgroup difference was 0.02. Table S6 summarizes meta‐analyses for nausea and vomiting separately and at different time points.

There was no difference in pain early postoperatively (0–6 h) nor late postoperatively (2–24 h), mean difference 0.0 (95% CI: −0.1, 0.0) and mean difference − 0.1 (95% CI: −0.3, 0.1), Table S6.

Details of the GRADE evaluation are provided in Table S7. The certainty in the evidence was assessed as very low for PONV and low to moderate for postoperative pain.

### 3.2 End‐tidal or arterial carbon dioxide level

Seven publications investigated different end‐tidal or arterial carbon dioxide level.^32^, ^33^, ^34^, ^35^, ^36^, ^37^, ^38^ Four trials compared P_et_CO_2_ levels ranging from 26 to 50 mmHg.^34^, ^35^, ^36^, ^38^ Two trials compared P_a_CO_2_ levels ranging from 35 to 65 mmHg. One trial compared an individualized P_et_CO_2_ with a fixed P_et_CO_2_.^33^ Additional information of the trials, including bias assessment, is reported in Tables S8–S11. Besir et al. found beneficial effects of hypocapnia on PONV in laparoscopic gynecological operations, whereas Saghaei et al. found a protective effect of mild hypercapnia on PONV in percutaneous nephrolithotomy operations.^35^, ^38^ Due to the heterogeneity in the intervention and the small number of trials, meta‐analyses were not considered meaningful.

### 3.3 Tidal volume and positive end‐expiratory pressure

Six publications investigated the effect of different tidal volumes.^39^, ^40^, ^41^, ^42^, ^43^, ^44^ The trials were grouped in higher (9–12 ml/kg) vs. lower tidal volumes (5–7 ml/kg). Five of the trials included an additional intervention of high positive end‐expiratory pressure (PEEP 6–10 cm H_2_0 vs. PEEP 0–4 cm H_2_0).^39^, ^41^, ^42^, ^43^, ^44^ Two of the trials included recruitment maneuvers in the intervention group.^39^, ^43^ Additional information is reported in Tables S12–S15.

Three trials investigated high (5–12 cm H_2_0) vs. low PEEP (0–4 cm H_2_0), one including recruitment maneuvers.^45^, ^46^, ^47^ Bluth et al. included 989 patients; the two other trials included ≤150 patients.

None of the six trials found a difference in postoperative pain. Due to the heterogeneity in type of surgery and intervention, no meta‐analysis was conducted for postoperative pain.

PONV was not reported in any of the trials regarding tidal volume or positive end‐expiratory pressure.

### 3.4 Recruitment maneuver

Eleven publications investigated the effect of recruitment maneuvers on pain and/or PONV.^48^, ^49^, ^50^, ^51^, ^52^, ^53^, ^54^, ^55^, ^56^, ^57^, ^58^ All trials included ≤150 patients with all patients undergoing surgery in the abdominal region. Ten of the trials compared recruitment maneuvers consisting of 2–6 manual pulmonary inflations with pressure up to 60 cmH_2_O with no recruitment maneuvers. The patients were placed in the Trendelenburg position during the maneuver, except in one trial with the patients in the supine position.^53^ One small trial compared recruitment maneuvers of different pressure, 15 vs. 30–40 cmH_2_O and was not considered further.^56^ Additional information is reported in Tables S16–S19.

**FIGURE 2:**
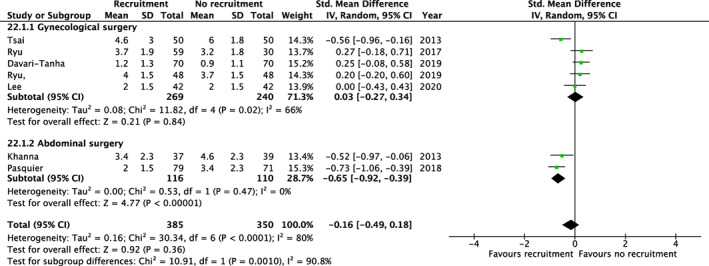
Recruitment maneuvers and pain 24 h postoperatively. Results from random‐effects meta‐analyses of trials assessing recruitment maneuvers. Results are displayed as mean differences (dots) with 95% confidence intervals (error bars). Values below 0 indicate reduced pain 24 h postoperatively with recruitment maneuvers.

In a meta‐analysis including seven trials and 735 patients, there was no clear difference in pain 24 h postoperatively (mean difference: −0.2, 95% CI: −0.5, 0.2) with recruitment maneuvers vs. no recruitment maneuvers, Figure 2. In a subgroup analysis, a significant reduction of pain was seen when considering the trials including abdominal surgery (mean difference −0.7, 95% CI: −0.9, −0.4), whereas no significant reduction of pain was found in gynecological surgery patients (mean difference: 0.0, 95% CI: −0.3, 0.3). The *p* value for the subgroup difference was 0.001. No significant difference in pain was found 48 h postoperatively, Figure S2.

**FIGURE 3:**
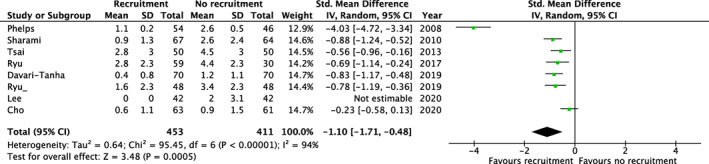
Recruitment maneuvers and shoulder pain 24 h postoperatively. Results from random‐effects meta‐analyses of trials assessing recruitment maneuvers. Results are displayed as mean differences (dots) with 95% confidence intervals (error bars). Values below 0 indicate reduced shoulder pain 24 h postoperatively with recruitment maneuvers.

Only laparoscopic gynecological surgery trials specifically investigated shoulder pain. In a meta‐analysis including nine trials and 864 patients, there was a significant reduction in shoulder pain 24 h postoperatively in the intervention group (mean difference: −1.1, 95% CI: −1.7, −0.5), Figure 3. Furthermore, in five of the trials including 500 patients the reduction in shoulder pain was maintained in the intervention group 48 h postoperatively (mean difference −0.8, 95% CI −1.0, −0.6), Figure S3. Results for shoulder pain at 4–6 h postoperatively is presented in Figure S4.

In a meta‐analysis including six trials and 620 patients, there was no difference in PONV (OR: 0.72, 95% CI: 0.31, 1.71), Figure S5.

Details of the GRADE evaluation are provided in Table S20. The certainty in the evidence was very low for pain, low for shoulder pain, and low for PONV.

### 3.5 Goal‐directed hemodynamic therapy

Sixteen publications investigated the effect of GDHT on pain and/or PONV, Tables S21–S24.^59^, ^60^, ^61^, ^62^, ^63^, ^64^, ^65^, ^66^, ^67^, ^68^, ^69^, ^70^, ^71^, ^72^, ^73^, ^74^

**FIGURE 4:**
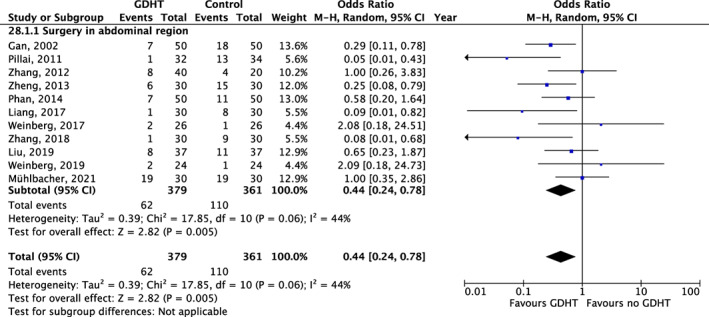
Goal‐directed hemodynamic therapy and postoperative nausea and vomiting. Results from random‐effects meta‐analyses of trials assessing goal‐directed hemodynamic therapy. Results are displayed as odds ratios (dots) with 95% confidence intervals (error bars). Values below 1 indicate reduced postoperative nausea and vomiting with goal‐directed hemodynamic therapy.

In a meta‐analysis including eleven trials and 740 patients, GDHT resulted in a significantly lower risk of PONV (OR: 0.44, 95% CI 0.24, 0.78), Figure 4. All but one trial included patients undergoing surgery in the abdominal region. When excluding the trial with surgery in the central nervous system, GDHT still resulted in a significant lower risk of PONV (OR: 0.48, 95% CI: 0.27, 0.85).

Pain was only reported in two trials.^63^, ^73^

Details of the GRADE evaluation are provided in Table S25. The certainty in the evidence was very low for PONV.

### 3.6 Other interventions

Two publications investigated the effect on pain of different blood pressures.^75^, ^76^ Delfino et al. found reduced pain score with mild hypertension during laparoscopic cholecystectomy, whereas the other trial found no difference. Two publications reported the effect on pain of different ventilation modes and found no difference in postoperative pain.^77^, ^78^ These trials are only reported descriptively (Tables S1 and S26–S29).

## 4 DISCUSSION

In this manuscript, we have systematically identified and described clinical trials assessing various intra‐operative respiratory and hemodynamic interventions reporting pain, nausea, and/or vomiting as outcomes. The main conclusions are: (1) Many trials did not report postoperative pain, nausea, or vomiting as an outcome or only reported these outcomes as secondary findings, (2) most of the included trials were small, (3) there was heterogeneity in the included trials limiting the interpretability of meta‐analyses, and (4) despite some positive findings there is limited evidence to support that intraoperative respiratory or hemodynamic interventions can meaningfully influence postoperative pain, nausea, and vomiting.

Postoperative pain, nausea, and vomiting are important outcomes for multiple reasons. First, these outcomes are by themselves important to patients. Avoidance of pain and PONV are important for a satisfactory recovery.^79^ Second, the development of pain and PONV are associated with an increased risk of complications and prolonged hospital stay.^1^, ^2^ Given these factors, it seems relevant to collect and report these outcomes for trials evaluating intra‐operative interventions. The current review shows that pain and PONV were infrequently and inconsistently reported.

A major limitation to the literature was the infrequent and inconsistent reporting of prophylaxis and treatment for PONV and pain. Given the unblinded nature of the various interventions, it is possible that such treatments could have differed between groups, potentially resulting in bias. Given this concern, all trials were assessed as having an intermediate risk of bias.

Exploratory meta‐analyses were conducted when considered feasible, that is, when there was a sufficient number of trials for a given treatment and outcome and when the trials were considered comparable. Although most of the analyses found no clear relationship between the intra‐operative intervention and the development of postoperative pain or PONV, we did identify some positive findings. There was some indication that a high FiO_2_ decreased PONV, especially in patients undergoing abdominal surgery. However, the certainty in the evidence was very low. Similarly, we found that the use of GDHT resulted in lower odds of PONV. The certainty of this evidence was also rated as very low. Given this, additional trials are needed before a definitive effect can be concluded. Moreover, it is important to consider these results in the context of other outcomes, such as mortality, length of stay, and postoperative complications as reported previously.^4^, ^6^

There was limited evidence that any of the intra‐operative hemodynamic or respiratory interventions affected postoperative pain. The one exception was the use of recruitment maneuvers at the end of laparoscopic gynecological surgery to reduce postoperative shoulder pain. This finding is consistent with previous reviews and the mechanism is considered to involve removal of air from the abdomen.^80^ However, the average effect was relatively small corresponding to a difference of approximately 1 on a pain scale from 0 to 10 and the certainty in the evidence was low. Although the minimally clinical important difference in pain varies substantially depending on the setting, most studies consider a change of 1 near or below the minimally clinical important difference.^81^ However, given the safety of recruitment maneuvers, this intervention can be considered.^5^

This systematic review has multiple strengths. We provide a comprehensive overview of hemodynamic and respiratory interventions that might affect postoperative pain and PONV. Trials were identified based on a broad search. The review follows recommended methodology including bias assessment and GRADE evaluation. There are also certain limitations. The review was limited to English language articles and relevant non‐English articles may be missed. The low number of relevant trials and heterogeneity among trials limited the conduct of meta‐analyses. Some articles only reported limited information on prophylactic PONV medication and treatment strategies for PONV and pain. The current manuscript reports multiple comparisons and there is therefore a risk of Type I errors. The specific outcomes reported in this manuscript were not prespecified in the protocol. Given these limitations, our findings should be considered hypothesis‐generating and not definitive.

In conclusion, although the overall certainty in the evidence was generally low, there was some indication that a high FiO_2_ and the use of GDHT might result in lower odds of PONV. Although most interventions did not affect pain, the use of recruitment maneuvers at the end of laparoscopic gynecological surgery likely results in a small reduction in postoperative shoulder pain. The results should be considered exploratory given the lack of prespecified hypotheses and corresponding risk of Type 1 errors. More definitive trials are needed to guide clinical care within this area.

## AUTHOR CONTRIBUTIONS

Lars W. Andersen, Mathias J. Holmberg, and Asger Granfeldt designed the review. All authors substantial contributed to the acquisition of the data. Johanne M. Holst and Lars W. Andersen analyzed the data. Johanne M. Holst and Lars W. Andersen drafted the manuscript and all authors revised it critically. All authors approved the final manuscript and agreed to be accountable for all aspects of the work.

## FUNDING INFORMATION

None.

## CONFLICT OF INTEREST

The authors declare no conflicts of interest.

## Supporting information


**Appendix S1** Supporting Information

## Data Availability

The datasets used are based on publicly available data from the included studies.
