# Supplementary material for: Intraoperative respiratory and hemodynamic strategies for reducing nausea, vomiting, and pain after surgery: Systematic review and meta‐analysis
Source: Acta Anaesthesiol Scand. 2022 Aug 22;66(9):1051–60. doi: 10.1111/aas.14127 (PMC9545575; doi:10.1111/aas.14127)
Supplement: Supplementary file 1 — Appendix S1 Supporting Information [file AAS-66-1051-s001.docx]

SUPPLEMENTARY CONTENT

**Intraoperative Respiratory and Hemodynamic Interventions for Reducing**

**Nausea, Vomiting, and Pain after Surgery: Systematic Review and Meta-Analysis**

CONTENT

Supplemental Methods 4

Definition of timeframes 4

Risk of bias assessment 4

GRADE evaluation 6

Supplemental Figure 1. Flow diagram of included trials. 8

Supplemental Table 1. Overview of the included trials 9

Fraction of inspired oxygen 16

Supplemental Table 2. Patient and surgical characteristics - fraction of inspired oxygen 16

Supplemental Table 3. Antiemetic and analgesia treatment - fraction of inspired oxygen 18

Supplemental Table 4. Outcome - fraction of inspired oxygen 20

Supplemental Table 5. Bias assessment - fraction of inspired oxygen 21

Supplemental Table 6: Results from meta-analyses 22

Supplemental Table 7. GRADE - fraction of inspired oxygen 23

End-tidal or arterial carbon dioxide level 24

Supplemental Table 8. Patient and surgical characteristics - end-tidal or arterial carbon dioxide level 24

Supplemental Table 9. Antiemetic and analgesia treatment - end-tidal or arterial carbon dioxide level 25

Supplemental Table 10. Outcome - end tidal or arterial carbon dioxide level 26

Supplemental Table 11. Bias assessment - end-tidal or arterial carbon dioxide level 27

Tidal volume and positive end-expiratory pressure 28

Supplemental Table 12. Patient and surgical characteristics - tidal volume and positive end-expiratory pressure 28

Supplemental Table 13. Antiemetic and analgesia treatment - tidal volume and positive end-expiratory pressure 29

Supplemental Table 14. Outcome - tidal volume and positive end-expiratory pressure 30

Supplemental Table 15. Bias assessment - tidal volume and positive end-expiratory pressure 31

Recruitment manoeuvre 32

Supplemental Table 16. Patient and surgical characteristics - recruitment maneuver 32

Supplemental Table 17. Antiemetic and analgesia treatment - recruitment maneuver 33

Supplemental Table 18. Outcome - recruitment maneuver 34

Supplemental Table 19. Bias assessment - recruitment maneuver 35

Supplemental Figure 2: Recruitment maneuver and pain 48 hours postoperatively 36

Supplemental Figure 3: Recruitment maneuver and shoulder pain 48 hours postoperatively 36

Supplemental Figure 4: Recruitment maneuver and shoulder pain 4-6 hours postoperatively 37

Supplemental Figure 5: Recruitment maneuver and postoperatively nausea and vomiting 37

Supplemental Table 20. GRADE - recruitment maneuver 38

Goal-directed hemodynamic therapy 39

Supplemental Table 21. Patient and surgical characteristics - goal-directed hemodynamic therapy 39

Supplemental Table 22. Antiemetic and analgesia treatment - goal-directed hemodynamic therapy 41

Supplemental Table 23. Outcome - goal-directed hemodynamic therapy 43

Supplemental Table 24. Bias assessment - goal-directed hemodynamic therapy 44

Supplemental Table 25. GRADE - Goal-directed hemodynamic therapy 45

Other interventions 46

Supplemental Table 26. Patient and surgical characteristics - other interventions 46

Supplemental Table 27. Antiemetic and analgesia treatment - other interventions 47

Supplemental Table 28. Outcome - other interventions 48

Supplemental Table 29. Bias assessment - other interventions 49

PRISMA-checklist 50

References 53

# Supplemental Methods

### Definition of timeframes

Various timeframes were reported in the publications. The most commonly used timeframes were selected:

*Fraction of inspired oxygen:*

Early (0-2h, 0-4h, 0-6h), late (2-24h, 6-24h, next morning), total (24h, any)

*End-tidal or arterial carbon dioxide level:*

Early (0-1h, 0-2h), late (2-6h, 2-24h), total (0-24h, next morning, any)

*Tidal volume, positive end-expiratory pressure and recruitment maneuver:*

Early (0h, 4h, 6h), late (24h, 1^st^ day), later (48h, 2^nd^ day, any)

*Goal-directed hemodynamic therapy:*

Early (0-6h), later (6-24h, 1^st^ day), total (total, later)

### Risk of bias assessment

Risk of bias was assessed using version 2 of the Cochrane Risk-of-Bias tool for individually-randomized parallel-group trials.^1^ Some general considerations related to this specific review are provided below.

*Risk of bias arising from the randomization process*

Three elements are considered within this domain: random allocation sequence, allocation concealment, and baseline imbalances. In general, if randomization was described and there was no indication of loss of allocation concealment, the trial was categorized as low risk of bias. This included trials with no clear description of allocation concealment as it was then assumed to be present. If the randomization process was not described at all, the trial was generally considered to be at an intermediate risk of bias.

*Risk of bias due to deviations from the intended interventions*

This domain focuses on whether participants received the intended intervention without other differences in care. As such, the domain primarily focuses on blinding of participants and the clinical team. By the nature of the various strategies, it is generally impossible to blind the clinician who provides the intervention, and for much of the treatment data, it is infeasible to decipher whether differences result from the intervention or non-protocol treatments. Therefore all trials were judged to be at least intermediate risk of bias.

The difference between the effect of assignment to an intervention and the effect of adherence to an intervention was difficult to assess and often not described in the trials. Although the effect of adherence to the intervention was technically the effect of interest, the distinction between the two was not considered further. Since appropriate per-protocol analyses were rarely performed, when possible, results from (modified) intention-to-treat analyses were included.

*Risk of bias due to missing outcome data*

For most trials, outcomes were only assessed in-hospital, and the amount of missing data was low. The trials were therefore classified as having a low risk of bias. In a few trials, loss to follow-up was more substantial. When this loss to follow-up was moderate and equal among groups, the risk of bias was assessed as intermediate; if the loss to follow-up was more substantial and different between groups, the risk of bias was classified as high.

*Risk of bias in measurement of the outcome*

For most trials, the methods of measuring the outcomes were described very limited. If the patients were blinded to the intervention, the risk of bias was assessed as low. Otherwise, it was assessed as intermediate. It was considered unlikely that knowledge of the intervention would have a major impact on the measurement of the outcome.

*Risk of bias in selection of the reported result*

If the trials reported results consistent with a protocol or trial registration, this domain was classified as low risk of bias. If there was no protocol or trial registration, the trial was classified as intermediate risk of bias. Outcome-relevant discrepancies between the manuscript and the protocol or trial registration were classified as intermediate or high based on whether or not it was selected from multiple outcome measurements or analyses of the data.

*Overall risk of bias*

The overall risk of bias was generally based on the highest risk reported within an individual domain.

### GRADE evaluation

The GRADE methodology was used to evaluate the overall certainty in the evidence for a given intervention and outcome. The following domains were considered.

*Risk of bias*

Risk of bias was rated as “serious” for all interventions and outcomes since almost all of the individual trials were assessed as having an intermediate risk of bias.

*Inconsistency*

Inconsistency was evaluated with the I^2^ statistic as well as visual inspection of the forest plot. As a guide, inconsistency was considered to the “serious” if the I^2^ statistic was > 40% but this also depended on the distribution of the individual trials.

*Indirectness*

Given that all the trials met the predefined criteria for inclusion and therefore represent the patient population of interest, indirectness was considered “not serious”. However, if the interest was in specific patient populations defined by patient characteristics or surgery type, it could be considered to assess indirectness as “serious”.

*Imprecision*

In determining whether a given comparison and outcome should be downgraded for imprecision, both the width of the confidence interval and the sample size (in that order) was considered. For binary outcomes, the effect was mainly considered on the relative scale (*i.e.*, odds ratio) but also evaluated on the absolute scale *(i.e.,* risk difference). If the confidence interval was very wide (*i.e.* <0.50 and >2.00 for the odds ratio) and included both potential benefit and harm, the imprecision was considered to be “very serious”. If the confidence was less wide and included potential benefit and no clear effect or harm, imprecision was considered “serious”. If the confidence interval only included clear benefit or harm or was narrow around no effect (*i.e.,* within 0.80 to 1.20 for the odds ratio), the sample size was considered in relation to the optimal information size.^2^ For pain, a narrow confidence interval was considered within -1 to 1 days on a 0 to 10 pain scale.

The optimal information size is equivalent to the required sample size of a single, adequately powered trial. It is challenging to determine a general optimal information size. However, considering an absolute risk reduction of 1%, the optimal information size would be 6206 for a control group outcome proportion of 2% and 39,494 for a control group outcome proportion of 2%. If a 5% absolute risk reduction were considered, the optimal information size would be 1164 for a control group outcome proportion of 10% and 2424 for 20%. These calculations are based on a chi-squared test, an alpha of 5%, and 90% power. Based on these considerations and previous suggestions^2^, the imprecision was considered to be “serious” if the sample size was less than 2000 patients. If the sample size was less than 100 patients or there were less than 10 events, the imprecision was considered to be “very serious”.

*Others*

In this domain, multiple aspects were considered including publication bias, the size of the effect, the direction of potential bias, and dose response gradients. The last two were not of relevance in the current review. Given the small number of trials, it was not feasible to evaluate for publication bias. A large effect was considered to be <0.50 or >2.00 on the odds ratio scale. For pain, a large effect was considered to be >3 on a 0 to 10 scale. Trials were only upgraded for a large effect if there was not very serious imprecision.

# Supplemental Figure 1. Flow diagram of included trials.

aPubMed
(n = 14 559)

EMBASE
(n = 21 590)

Total number of records identified
(n = 36 149)

Records screened
(n = 23 454)

Records excluded (Kappa = 0.61)
(n = 22 919)

Full-text articles assessed for eligibility
(n = 535)

Full-text articles excluded
(n = 472)

Not relevant outcome = 336

No relevant intervention = 51

Other = 28

One lung ventilation = 15

Obstetrics = 11

Not general anesthesia = 8

Cross over study = 7

Missing full texts = 5

No relevant study type = 5

Pediatric = 3

Very short duration of anesthesia = 2

Invasive radiology = 1

Manuscripts included
for the review

(n = 65)

Duplicates excluded
(n = 12 695)

Identified in bibliographies

(n = 2)

| Supplemental Table 1. Overview of the included trials | | | | | | |
| --- | --- | --- | --- | --- | --- | --- |
| **Trial: first author, publication year** | **Country** | **Years of Inclusion** | **Inclusion criteria** | **Exclusion criteria** | **Comparator** | **Intervention** |
| **Category: Fraction of inspired oxygen** | | | | | | |
| **Kurz, 2015**^3^ | Multiple | 2002-2007 | Age ≤80, elective colorectal resection expected to last 2-6 hours | Chemotherapy within 6 months, anticipated secondary wound closure, fever or infection, severe COPD, unstable angina pectoris, hypertensive cardiomyopathy, congestive heart failure, MI, bowel obstruction, ASA ≥IV | FiO_2_ 30% | FiO_2_ 80% |
| **McKeen, 2015**^4^ | Canada | NR | Ambulatory laparoscopic tubal ligation, ASA I-II | Preoperative benzodiazepines, diabetes, gastro paresis, BMI >37, breastfeeding, nausea/vomiting or antiemitic therapy, current opioid/cannabis use, known substance abuse, previous severe PONV | FiO_2_ 30% | FiO_2_ 80% |
| **Simurina, 2009**^5^ | Croatia | NR | ASA I-II, elective gynecologic laparoscopic surgery | BMI >30, pulmonary diseases, pregnancy or breast feeding, known hypersensitivity to drugs used in the study protocol, use of antiemetics, psychotropic drugs, hormone and steroid use within three days of surgery, diseases that impaired gastric motility, patients with vestibular disease, history of migraine headache, central nervous system injury, renal impairment, irregular menstrual cycles, alcoholism, opioid addiction | FiO_2_ 30% | FiO_2_ 80%/  FiO_2_ 50% |
| **Goll, 2001**^6^ | Austria | NR | Age 19-70, ASA I-II, gynecological laparoscopy, expected surgical duration >1 hour, overnight hospitalization | Pregnancy, breast-feeding, menopausal status, >150% normal weight, eating disorder, renal or liver malfunction, CNS injury, vertebrobasilar artery insufficiency, vestibular disease, cytostatic therapy, preoperative vomiting or antiemetic therapy | FiO_2_ 30% | FiO_2_ 80% |
| **Joris, 2003**^7^ | Belgium | NR | Age 19-70, ASA I-III, elective thyroid surgery | >150% ideal body weight, gastro-esophageal reflux, history of motion sickness, previous PONV, preoperative vomiting, antiemetic therapy, pulmonary disease. | FiO_2_ 30% | FiO_2_ 80% |
| **Mraovic, 2008**^8^ | Croatia | NR | ASA I-II, age 18-75, elective laparoscopic gynecological surgery (ovarian tumour/cyst removal, myomectomy, hysterectomy, infertility surgery) | BMI >33, pregnancy, breast-feeding, known hypersensitivity to drugs used in study, use of antiemetics, psychotropic drugs and steroids within 72 hours of surgery, diabetes mellitus, chronic cholecystitis, GI-disease, neuromuscular disease, neuropathies, liver dysfunction, vestibular disease, migraine, CNS injury, renal impairment, irregular menstrual cycle, alcoholism, opioid addiction | FiO_2_ 30% | FiO_2_ 50% + N_2_O/  FiO_2_ 30% +  N_2_O |
| **Purhonen, 2006**^9^ | Finland | 2003-2004 | Female, age 18-75, ASA I-III, breast surgery | Nausea or vomiting within 24 hours, antiemetic drugs within 24 hours, pregnancy or breastfeeding, BMI >30, abuse of alcohol or drugs, diabetes, pulmonary disease | FiO_2_ 30% | FiO_2_ 80% |
| **Purhonen, 2003**^10^ | Finland | NR | Female, ASA I-II, ambulatory gynecologic laparoscopy | Nausea or vomiting within 24 hours, antiemetic drugs within 24 hours, pregnancy or breastfeeding, BMI >30, pulmonary disease | FiO_2_ 30% | FiO_2_ 80% |
| **Thibon, 2012**^11^ | France | 2003-2007 | Elective abdominal, gynecological, or breast surgery | Fever, infection, chronic respiratory failure, Bleomycin treatment | FiO_2_ 30% | FiO_2_ 80% |
| **Treschan, 2005**^12^ | Germany | NR | Age 5-79, ASA I-II, strabismus surgery | Predisposition for malignant hyperthermia, previous antiemetic treatment, psychiatric disorders, pregnancy | FiO_2_ 30% | FiO_2_ 80% |
| **Purhonen, 2003**^13^ | Finland | NR | ASA I-III, female, scheduled for breast surgery | NR | FiO_2_ 30% | FiO_2_ 50% |
| **Sadrolsadat, 2007**^14^ | Iran | 2005-2006 | ASA I, male, age 20-50, inguinal hernia surgery | History of cigarette smoking, substantial alcohol use, obesity (>150% normal weight), eating disorder, renal or liver malfunction, central nervous system injury, vertebrobasilar artery insufficiency, vestibular disease, cytostatic therapy, preoperative vomiting or antiemetic therapy | FiO_2_ 30% | FiO_2_ 70% |
| **Alvandipour, 2018**^15^ | Iran | NR | Colorectal surgery | COPD, pneumonia, BMI >35, severe malnutrition, leukopenia, surgery within the previous 30 days, O_2_ saturation < 90% | FiO_2_ 30% | FiO_2_ 80% |
| **Meyhoff, 2009**^16^ | Denmark | 2006-2008 | Abdominal laparotomy | Other surgery within 30 days, chemotherapy within 3 months, SpO_2_ <90% without supplemental oxygen. | FiO_2_ 30% | FiO_2_ 80% |
| **Myles, 2007**^17^ | Multiple | 2003-2004 | Expected duration >2 hours, anticipated length of hospital stay ≥3 days | Cardiac surgery, one-lung ventilation, contraindication to nitrous oxide | FiO_2_ 30% + N_2_O | FiO_2_ 80% |
| **Turan, 2006**^18^ | Multiple | 2000-2002 | Elective surgery under GA, expected duration >1 hours, ≥40% risk of PONV | Study drugs contraindicated, emetogenic or anti-emetic drugs within the 24 hours, expected postoperative mechanical ventilation, pregnant or lactating | FiO_2_ 30% | FiO_2_ 80% |
| **Cohen, 2018**^19^ | United States of America | 2013-2016 | Colorectal surgery | Missing data, duration of surgery <2h, reoperation during the same visit AKI substudy: severe chronic kidney disease, renal transplant, sepsis, eGFR >15 mL/min. | FiO_2_ 30% | FiO_2_ 80% |
| **Li, 2020**^20^ | China | 2018 | >=18 years, ASA I-III, elective abdominal surgery, expected surgical duration >2h, planned to be extubated in the operating room | History of acute lung injury or acute respiratory distress syndrome in last three months, heart failure (NYHA IV), chronic renal failure (glomerularfiltration rate <30 ml), serious hepatic diseases (e.g.,hepatic failure), pregnancy, expecting to receive reoperation or post-operative mechanical circulatory support, consent for other trials, BMI >30 | FiO_2_ 30% | FiO_2_ 80% |
| **Category: End-tidal or arterial carbon dioxide level** | | | | | | |
| **Wang, 2019**^21^ | China | 2016-2017 | ASA II-III, laparoscopic surgery for adenocarcinoma of the rectum | Second primary malignancy, severe cardiac dysfunction, intracranial disease, mental disorder, visual or auditory dysfunction, prior therapies for rectal cancer, severe COPD, acute inflammatory bowel disease, obesity, pregnancy, surgery >5 hours, conversion to laparotomy | PaCO_2_ 3.5-4.5 | PaCO_2_ 4.6-5.5/  PaCO_2_ 5.6-6.5 |
| **Mäkinen, 2000**^22^ | Finland | NR | ASA I-II, elective laparoscopic cholecystectomy | Respiratory disease or BMI >30 | Fixed ventilation | P_et_CO_2_ 4.5 |
| **Murphy, 2014**^23^ | United States of America | NR | ASA I-III, elective shoulder arthroscopy, beach chair position, age 18-80 | Orthostatic hypotension, poorly controlled hypertension, pre-existing history of cerebrovascular or pulmonary disease, symptomatic cardiovascular disease | P_et_CO_2_ 3.0-3.2 | P_et_CO_2_ 4.0-4.2 |
| **Saghaei, 2014**^24^ | Iran | 2012 | Percutaneous nephrolithotomy, "otherwise healthy" | History of PONV, recent general anesthesia with volatile anesthetics, cardio-respiratory diseases, elevated blood urea nitrogen or creatinine, addiction, smoking, BMI >30 | P_et_CO_2_ 3.7-3.9 | P_et_CO_2_ 3.1-3.3/  P_et_CO_2_ 4.3-4.5 |
| **Akça, 2013**^25^ | Multiple | 2002-2007 | Age ≤-80, elective colorectal resection expected to last 2-6 hours | Chemotherapy within 6 months, anticipated secondary wound closure, fever or infection, severe COPD, unstable angina pectoris, hypertensive cardiomyopathy, congestive heart failure, MI, bowel obstruction, ASA ≥IV | P_et_CO_2_ 3.5 | P_et_CO_2_ 50 |
| **Son, 2017**^26^ | South Korea | NR | Age 20-60, ASA I-II, laparoscopic gynecologic surgery, anticipated use of opioids postoperatively | Motion sickness or PONV, diabetes, COPD, GI disease, smokers, actively menstruating, BMI <16 or >35, use of antiemetics or steroids within 72 hours prior to surgery | PaCO_2_ 3.6-4.0 | PaCO_2_ 4.1-4.5/  PaCO_2_ 4.6-6.0 |
| **Besir, 2020**^27^ | Turkey | 2018 | Female, ASA I-II, gynecologic laparoscopic surgery, age 25-50, non-smoker, 2 of 4 PONV score | Ophthalmic diseases or surgery, neurologic diseases increasing ICP, PONV, smoking habit, car sickness, hepatic and renal dysfunction, abnormal fluid-electrolyte balance, preoperative antiemetic drug use, severe heart failure, tumors of orbit, arrhythmia | P_et_CO_2_ 2.6-3.5 | P_et_CO_2_ 3.6-4.5 |
| **Category: Tidal volume and positive end-expiratory pressure** | | | | | | |
| **Asida, 2015**^28^ | Egypt | 2013 | Elective non-laparoscopic urological operations >2 hours, right or lateral position, normal respiratory, hepatic and cardiac function, hemodynamically stable | BMI>30, history of COPD, asthma, sleep disorders, heavy smokers (>2 packs/day), previous lung surgery, acute lung injury, history of neuromuscular disease, on medication with effect in respiratory system | TV 10-12  PEEP 0 | TV 5-7  PEEP 10  2 RM |
| **Severgnini, 2013**^29^ | Italy | 2006-2008 | Elective open abdominal surgery, expected duration >2 hours | BMI >40, previous lung surgery, persistent hemodynamic instability, intractable shock, COPD, asthma, sleep disorder, recent chemotherapy or radiation therapy, NYHA III-IV, acute coronary syndrome, persistent ventricular tacky-arrhythmias, pregnancy, acute lung injury, ARDS, expected to require prolonged postoperative mechanical ventilation, any neuromuscular disease, contraindications of an epidural catheter, sign of infection at the site of the procedure | TV 9  PEEP 0 | TV 7  PEEP 10  2 RM |
| **Haliloglu, 2017**^30^ | Turkey | 2011 | ASA I-II, robot-assisted laparoscopic radical prostatectomy | Cardiovascular and respiratory diseases, BMI >40 | TV 10  PEEP 0 | TV 6  PEEP 8 |
| **Mølsted, 2020**^31^ | Denmark | NR | Elective robot-assisted radical prostatectomy | Severe renal failure, BMI >35, previous lung surgery, lung disease requiring pharmacological treatment, NYHA class III-IV, MI within 12 months, neuromuscular disease, intraoperative bleeding requiring transfusion, intraoperative hypoxemia, persistent plateau pressure > 30 cmH_2_O, conversion to open surgery, prolonged postoperative course due to re-operation or infection, poor compliance | TV 10  PEEP 4 | TV 6  PEEP 10 |
| **Shin, 2010**^32^ | South Korea | 2009 | ASA I-II, laparoscopic appendectomy | Use of analgesics within 24 hours before surgery, BMI >30 | TV 10 | TV 7 |
| **Soh, 2018**^33^ | South Korea | 2015-2016 | Major lumbar spinal surgery, expected duration >2 hours, high risk for pulmonary complications | Increased intracranial pressure, altered mental status, neuromuscular disease, BMI >35, previous lung surgery, repeated treatment for acute exacerbation of asthma or COPD, congestive heart failure, use of mechanical ventilation within 2 weeks before surgery, sepsis, pregnancy | TV 10  PEEP 6 | TV 6  PEEP 6 |
| **Bluth, 2019**^34^ | Multiple | 2014-2018 | BMI >35, surgery >2 hours, Assess Respiratory Risk in Catalonia score >26 | Age <18 years, previous lung surgery, mechanical ventilation >30 min within 30 days prior to surgery, cardiac or neurological surgery, one-lung ventilation, planned re-intubation after surgery, prone or lateral decubitus position during surgery, pregnant, intractable shock, severe COPD, severe cardiac disease, concurrent ARDS, severe pulmonary hypertension, intracranial tumor, neuromuscular disease | PEEP 4 | PEEP 12  RM |
| **Wetterslev, 2001**^35^ | Denmark | NR | Upper abdominal surgery, no significant pre-existing cardiopulmonary disease, BMI <34, ASA 1-2 | NR | PEEP 0 | PEEP 5, 8, or 10  (depending on static pulmonary compliance) |
| **Seo, 2018**^36^ | South Korea | 2015-2016 | ASA I-II, spine surgery in prone position | Obesity, thyroid disease, peripheral vascular disease, diabetes, uncontrolled hypertension, COPD, intraoperative position change, induced hypothermia for intraoperative neuroprotection, temperature of >38 or <35 | PEEP 0 | PEEP 10 |
| **Category: Recruitment manoeuvre** | | | | | | |
| **Cho, 2020**^37^ | South Korea | 2008-2009 | Elective gynecologic laparoscopic surgery for benign adnexal disease, age 15-65 | Conversion to laparotomy, operative time > 3 hours, serious adverse effects which made it impossible to score pain | No RM | RM |
| **Khanna, 2013**^38^ | United Kingdom | 2010-2012 | ASA I - II, elective laparoscopic cholecystectomy for gallbladder disease or laparoscopic inguinal hernia repair | Pregnancy, additional surgical procedures | No RM | RM |
| **Ryu, 2017**^39^ | South Korea | 2015-2016 | Age 19-65, ASA I-II, laparoscopic benign gynecologic surgery | Pregnancy, inability to express pain, concomitant surgery, previous shoulder or lung surgery, chronic shoulder problems or epigastric pain, lung diseases | No RM | RM  (40/60cm H_2_O) |
| **Sharami, 2010**^40^ | Iran | 2008-2009 | Age 15-50, no previous laparotomy, ASA I-II, minor laparoscopic gynecological surgery | Intra-abdominal pressure >15 mmHg during the operation | No RM | RM |
| **Davari-Tanha, 2019**^41^ | Iran | 2013-2015 | Age 20-65, benign gynecologic diseases, ASA I-II, laparoscopic gynecologic procedures | NR | No RM | RM |
| **Lee, 2020**^42^ | South Korea | 2018 | Age 19-65, ASA I-II, elective, benign gynecologic laparoscopy | Inability to express pain, pregnancy, former thoracic or shoulder surgery, pulmonary disease, chronic shoulder pain, concomitant abdominal surgery, conversion to laparotomy | No RM | RM |
| **Pasquier, 2018**^43^ | Sweden | 2014-2016 | BMI ≥35, ASA I-II, elective laparoscopic bariatric surgery | Conversion to open surgery, complications classified as Clavien-Dindo grade ≥II | No RM | RM |
| **Phelps, 2008**^44^ | United States of America | 2003-2005 | Elective outpatient gynecologic laparoscopic surgery, age 15-65, ASA I-II, no previous laparotomy | Conversion to laparotomy, 48-hour follow-up not feasible | No RM | RM |
| **Ryu, 2019**^45^ | South Korea | 2016-2017 | Age 19-65, ASA I - II, laparoscopic benign gynecologic surgery | Inability express pain, pregnancy, pulmonary disease, chronic shoulder pain, incidental upper abdominal procedures | No RM | RM |
| **Yilmaz, 2020**^46^ | Turkey | 2019 | ASA I-II, age 18-70, gynecologic laparoscopy for non-malignant conditions | Chronic shoulder and epigastrial pain, previous lung or shoulder surgery, chronic emphysema, pneumothorax, pregnancy, unable to express pain, candidate for potential concomitant upper abdominal surgery | RM | RM |
| **Tsai, 2013**^47^ | Taiwan | 2011-2012 | Female, age 20-65, ASA I-II, willing to undergo laparoscopic surgeries for benign gynecologic lesions | Malignant disease, unwillingness | No RM | RM |
| **Category: Goal-directed hemodynamic therapy** | | | | | | |
| **Bundgaard-Nielsen, 2013**^48^ | Denmark | 2008-2009 | Open radical prostatectomy | ASA >III, sedative premedication, psychiatric disease, alcohol abuse, kidney disease, coagulation impairment, opioid consumption, orthostatic hypotension, use of β-blockers, need for intraoperative infusion of vasopressor or inotropic agents other than ephedrine, contraindication to esophageal Doppler use | Standard care | GDHT |
| **Ramsingh, 2013**^49^ | United States of America | NR | Major open abdominal non-vascular surgery | Coagulopathy, cerebrovascular disease, renal or hepatic dysfunction, congestive heart failure, ischemic heart disease, cardiac arrhythmias, lunge disease, choice | Standard care | GDHT |
| **Liang, 2017**^50^ | China | 2014-2015 | Age 60-80, hypertension, transurethral resection of prostate due to benign prostatic hyperplasia, ASA II-III | Abnormal liver function, abnormal kidney function, BMI ≥30, perioperative hematocrit ≤ 0.30, secondary hypertension, prostate cancer, severe cardiopulmonary disease, arrhythmia | Standard care | GDHT |
| **Liu, 2019**^51^ | China | 2017-2018 | Age 65-80, ASA I-II, laparoscopic resection of colorectal cancer | Incompensated cardiac or respiratory disease, neurological disease, peripheral vascular disease, coagulopathy, anemia, renal impairment, previous major operation | Standard care | GDHT |
| **Luo, 2017**^52^ | China | NR | ASA III-IV, elective craniotomy for brain tumor resection, brain abscess, or intracranial aneurysm, expected duration >2 hours | Weight <40 or >100, arrhythmia | Standard care | GDHT |
| **Noblett, 2006**^53^ | United Kingdom | NR | Elective colorectal surgery | Severe esophageal disease, recent esophageal or upper airway surgery, use of systemic steroids, moderate to severe aortic valve disease, bleeding | Standard care | GDHT |
| **Zhang, 2018**^54^ | China | NR | Age >60, ASA I-II, BMI <30, hematocrit >0.35, surgery for spinal stenosis | Chronic disease, history of mental illness, neurological disease, receiving drugs that may affect cognitive function, arrhythmia, liver and kidney dysfunction, severe cardiopulmonary disease, low Montreal Cognitive Assessment Score | Standard care | GDHT |
| **Zheng, 2013**^55^ | China | 1999-2011 | GI surgery, age 60-80, prior coronary angiography, moderate to high-risk elective surgery, coronary heart disease, NYHA II-III, BMI 18-24, anticipated blood loss <600 mL, normal renal and liver function | ASA >III, prior fluid therapy within 48 hours, congenital heart disease, cardiomyopathy, rheumatic heart disease, pulmonary heart disease, preoperative use of vasoactive drugs >3 months, preoperative administration of diuretics, preoperative acid-base or electrolyte imbalance, difficulty placing central line, inability to cooperate, blood-borne infectious disease, undergone surgery twice after admission, dehydration | Standard care | GDHT |
| **Peng, 2014**^56^ | China | NR | Elective major orthopedic surgery, anticipated blood loss >800 ml | BMI >40 or <15, coagulopathy, arrhythmia or cardiopulmonary dysfunction, renal or liver disease | Standard care | GDHT |
| **Gan, 2002**^57^ | United States of America | NR | Major elective general, urologic or gynecologic surgery, anticipated blood >500 ml, ASA I-III | Preoperative bowel obstruction, coagulopathy, renal and hepatic dysfunction, congestive heart failure, esophageal pathology, gastric or esophageal surgery, antiemetic medication within 3 days of surgery | Standard care | GDHT |
| **Phan, 2014**^58^ | Australia | 2012-2013 | Major colorectal surgery | ASA IV, pregnancy, emergency surgery, renal dysfunction, hepatic dysfunction, NYHA III-IV, esophageal pathology | Standard care | GDHT |
| **Weinberg, 2017**^59^ | Australia | 2013-2015 | Elective pancreaticoduodenectomy | Pregnancy, coagulopathy, renal impairment, chronic liver disease, ASA V, distal, central or total pancreatectomy or pancreatic enucleation | Standard care | GDHT |
| **Weinberg, 2019**^60^ | Australia | 2013-2016 | Elective major liver resection | Coagulopathy, thrombocytopenia, renal impairment, hepatic insufficiency, ASA >III, EF <40%, atrial fibrillation, moderate or severe tricuspid regurgitation, impairment of right ventricular function | Standard care | GDHT |
| **Zhang, 2012**^61^ | China | NR | Age 18-64, anticipated blood loss <500 mL, gastric or colon cancer | BMI >30, significant arrhythmias, cardiopulmonary dysfunction, peripheral arterial occlusive disease, renal or liver disease, pregnancy, coagulopathy | Standard care | GDHT |
| **Mühlbacher, 2021**^62^ | Germany | NR | BMI >30, age 18-65, laparoscopic gastric bypass surgery | Decompensated heart failure, documented coronar or peripheral artery disease, renal insufficiency, severe COPD, insulin-dependent DM, known aortic or esophageal abnormality, obstructive sleep apnea requiring CPAP | Standard care | GDHT |
| **Pillai, 2011**^63^ | United Kingdom | 2007-2009 | Radical cystectomy for bladder cancer | Esophageal disease or recent esophageal or larynx surgery, moderate to severe aortic valve disease and patient choice | Standard care | GDHT |
| **Category: Other interventions** | | | | | | |
| **Delfino, 2015**^64^ | Chile | NR | ASA I-II, elective laparoscopic cholecystectomy, age 18-50 | Hypertension, baseline arterial pressure more than 139/89 mmHg, chronic pain, drug abuse, cerebrovascular disease, obesity (BMI >30), use of analgesic drugs and/or drugs acting on the CNS system, pregnancy, adverse effects to the study drugs | Systemic blood pressure <20-30% baseline | Systemic blood pressure >20-30% baseline |
| **Xu, 2020**^65^ | China | 2019 | Elective hip replacement, ASA II-III, age 65-80 | Brain tumor, previous cerebrovascular emergency, mental disease and use of psychotropic drugs within 6 months, communication disorder, liver and kidney dysfunction, long-term alcohol abuse, preoperative cognitive dysfunction | Mean arterial pressure <10 - 20% baseline | Mean arterial pressure <0 - 10% baseline |
| **Kim, 2019**^66^ | South Korea | 2017-2018 | Age 65-90, ASA I-III, BMI ≤31, no acute respiratory disease, hip joint surgery | Mental retardation or severe cognitive impairment, previous intrathoracic procedure, cardiopulmonary compromised status | VCV | PCV-VG |
| **Le Guen, 2019**^67^ | France | 2013-2015 | Elective pituitary gland surgery, transsphenoidal access | Pregnancy, BMI >35, chronic respiratory disease, redo or emergent surgery, coagulation disorders, aesthetic or surgical complications except bleeding related complications | VCV | PCV |

*BMI: body mass index, ASA: American Society of Anesthesiologists, NR: not reported, TV: tidal volume, PEEP: positive end-expiratory pressure, RM: recruitment maneuver, GDHT: goal-directed hemodynamic therapy, VCV: volume-controlled ventilation, PCV-VG: pressure-controlled ventilation, VG: volume guaranteed, /: different interventions, COPD:, chronic obstructive pulmonary disease, PONV: postoperative nausea and vomiting, CNS: central nervous system, ARDS: acute respiratory distress syndrome*

*Age is reported in years, BMI in kg/m^2^, and P_a_CO_2_ and P_et_CO_2_ in mmHg*

# Fraction of inspired oxygen

| Supplemental Table 2. Patient and surgical characteristics - fraction of inspired oxygen | | | | | | | | | | | | | | | | | |
| --- | --- | --- | --- | --- | --- | --- | --- | --- | --- | --- | --- | --- | --- | --- | --- | --- | --- |
| **Trial** | **n** | **Patient characteristics** | | | | | | | **Surgical characteristics** | | | | | | | | |
|  |  | **Age** | **Sex**  **(% male)** | **BMI** | **ASA** | | | | **Surgery** | | | | | **Laparoscopic (%)** | **Acute (%)** | **Duration (min.)** | |
|  |  |  |  |  | **1** | **2** | **3** | **4** | **Ort.** | **Abd.** | **Gyn.** | **Uro.** | **Oth.** |  |  | **Anesthesia** | **Surgery** |
| **Kurz, 2015**^3^ | 586 | 53 | 50 | 27 | 14 | 60 | 26 | 0 | 0 | 100 | 0 | 0 | 0 | 32 | 0 | NR | 210 |
| **McKeen, 2015**^4^ | 304 | 36 | 0 | 26 | 72 | 28 | 0 | 0 | 0 | 0 | 100 | 0 | 0 | 100 | 0 | 44 | 22 |
| **Simurina, 2009**^5^ | 120 | 38 | 0 | 23 | NR | NR | 0 | 0 | 0 | 0 | 100 | 0 | 0 | 100 | 0 | 70 | 52 |
| **Goll, 2001**^6^ | 240 | 37 | 0 | 24 | 40 | 55 | 5 | 0 | 0 | 0 | 100 | 0 | 0 | 100 | NR | 88 | 60 |
| **Joris, 2003**^7^ | 150 | 49 | 16 | 25 | NR | NR | NR | 0 | 0 | 0 | 0 | 0 | 100 | NR | 0 | 116 | NR |
| **Mraovic, 2008**^8^ | 150 | 37 | 0 | 24 | 77 | 23 | 0 | 0 | 0 | 0 | 100 | 0 | 0 | 100 | 0 | 73 | 56 |
| **Purhonen, 2006**^9^ | 90 | 53 | 0 | 24 | NR | NR | NR | 0 | 0 | 0 | 0 | 0 | 100 | 0 | 0 | 128 | 99 |
| **Purhonen, 2003**^10^ | 100 | 37 | 0 | 24 | NR | NR | 0 | 0 | 0 | 0 | 100 | 0 | 0 | 100 | 0 | 43 | 31 |
| **Thibon, 2012**^11^ | 434 | 52 | 10 | 25 | 50 | 46 | 4 | 0 | 0 | 20 | 32 | 0 | 48 | 27 | NR | NR | 87 |
| **Treschan, 2005**^12^ | 210 | 31 | 44 | 24 | NR | NR | 0 | 0 | 0 | 0 | 0 | 0 | 100 | 0 | 0 | 52 | 27 |
| **Purhonen, 2003**^13^ | 100 | NR | 0 | NR | NR | NR | NR | 0 | 0 | 0 | 0 | 0 | 100 | 0 | 0 | NR | NR |
| **Sadrolsadat, 2007**^14^ | 100 | 36 | 100 | 26 | 65 | NR | NR | NR | 0 | 100 | 0 | 0 | 0 | NR | 0 | 69 | 54 |
| **Alvandipour, 2018**^15^ | 85 | 59 | 25 | 26 | NR | NR | NR | NR | 0 | 100 | 0 | 0 | 0 | 0 | 31 | NR | NR |
| **Meyhoff, 2009**^16^ | 1400 | 64 | 42 | 25 | 27 | 54 | 18 | 1 | 0 | 81 | 19 | 0 | 0 | 0 | 28 | 193 | 130 |
| **Myles, 2007**^17^ | 2050 | 55 | 52 | NR | 21 | 55 | 22 | 2 | 9 | 46 | 7 | 13 | 25 | NR | 4 | 222 | 198 |
| **Turan, 2006**^18^ | 560 | 43 | 36 | NR | NR | NR | NR | NR | NR | NR | NR | NR | NR | NR | NR | 138 | NR |
| **Cohen, 2018**^19^ | 4702 | 53 | 48 | 27 | NR | NR | 60 | NR | 0 | 100 | 0 | 0 | 0 | 30 | NR | NR | 230 |
| **Li, 2020**^20^ | 251 | 53 | 50 | 23 | 1 | 85 | 14 | 0 | 0 | 100 | 0 | 0 | 0 | 27 | 0 | 260 | 195 |

*n: sample size, BMI: body mass index, ASA: American Society of Anesthesiologists, Ort: orthopedic, Abd.: abdominal, Gyn.: gynecological, Uro.: urological, Oth.: other, NR: not reported*

| Supplemental Table 3. Antiemetic and analgesia treatment - fraction of inspired oxygen | | | | | | | | | |
| --- | --- | --- | --- | --- | --- | --- | --- | --- | --- |
| **Trial** | **Prophylactic antiemetic treatment**  **n/total (%)** | **Any rescue antiemetic treatment**  **n/total (%)** | | | **Epidural treatment**  **n/ total (%)** | | **PACU opioid analgesia use**  **mean (SD)** | | |
|  |  | **Comparator** | **Intervention** | **Note** | **Comparator** | **Intervention** | **Comparator** | **Intervention** | **Note** |
| **Kurz, 2015**^3^ | Dexamethasone 283/555 (51) | NR | NR | Not standardized  %Dexamethasone | 46/270  (17) | 37/285 (13) | NR | NR | Patient-controlled analgesia device with IV administration of morphine or hydromorphone |
| **McKeen, 2015**^4^ | 0 | 49/145 (34) | 43/147 (29) | Vomiting, nausea >15min: Ondansetron. Persistent symptoms: Diphenhydrinate and/or Dexamethasone | NR | NR | 10.0 (5.8) | 10.5 (6.4) | Morphine equivalens |
| **Simurina, 2009**^5^ | 0 | 8/36 (22) | 5/36 (14)/  5/36 (14) | Metoclopramide (=>2 episodes of vomiting or retching within a period of 30 minutes, nausea >15 minutes, a nausea VAS score N 50 mm, requested treatment) | NR | NR | 10.7 (23.6) | 7.0 (15.4)/  5.3 (11.8) | Meperidine, mg |
| **Goll, 2001**^6^ | 0 | 23/80 (29) | 11/79 (14) | Ondansetron (nausea >15-20 min or vomiting) | NR | NR | 2.9  (4.0) | 2.4  (3.9) | Piritramid, mg |
| **Joris, 2003**^7^ | Hydroxyzine 25 mg  150/150 (100) | 19/50 (38) | 21/50 (42) | Alizapride (upon request), tropisetron 2mg (PONV persisted for >30 min) | NR | NR | 4 (3) | 4 (2) | Piritramid, mg tramadol is mentioned as rescue, no numbers |
| **Mraovic, 2008**^8^ | 0 | 7/46 (15) | 10/46 (22)/  10/45 (22) | Metaclopramide (by request, >=episodes of POV within 30min, nausia >15min or severe nausea) | NR | NR | 6.5 (22.0) | 7.1 (16.4)/  10.1 (19.6) | Meperidine, mg |
| **Purhonen, 2006**^9^ | 0 | 17/28 (61) | 14/29 (48) | Nausea >15 min, for the second emetic episode, and at request. Droperidol (1), dexamethasone (2) and ondansetron (3) | NR | NR | 16  (9) | 16  (12) | Oxycodone IV or IM, mg |
| **Purhonen, 2003**^10^ | 0 | 10/50 (20) | 13/49 (27) | Nausea >15 min, second emetic episode, and at request. Ondansetron (1 and 2), droperidol (3) | NR | NR | 95  (107) | 77  (89) | Fentanyl, **μ**g |
| **Thibon, 2012**^11^ | NR | NR | NR |  | NR | NR | NR | NR |  |
| **Treschan, 2005**^12^ | 0 | 11/71 (15) | 8/68 (12) | Dimenhydrinate on request. Droperidol when persisting nausea/vomiting | NR | NR | NR | NR |  |
| **Purhonen, 2003**^13^ | NR | 25/49 (51) | 23/47 (49) | Ondansetron (1 and 2) and droperidol (3) | NR | NR | NR | NR | Oxycodone IM or IV |
| **Sadrolsadat, 2007**^14^ | 0 | NR | NR |  | NR | NR | NR | NR |  |
| **Alvandipour, 2018**^15^ | NR | NR | NR |  | NR | NR | NR | NR |  |
| **Meyhoff, 2009**^16^ | Dexamethasone  440/1386 (32)  Equal distributed | NR | NR |  | 498/701 (71) | 473/685 (69) | NR | NR |  |
| **Myles, 2007**^17^ | Equal distributed, no information about drug type | NR | NR |  | NR | NR | NR | NR |  |
| **Turan, 2006**^18^ | Randomized to ondansetron, dexamethason, droperidrol or control. Apparently equal distribution in groups | 3/279 (1) | 6/280 (2) | Ondansetron (1), dexamethasone (2), and droperidol (3). Further medication was the choice of the investigator | NR | NR | NR | NR |  |
| **Cohen, 2018**^19^ | NR | NR | NR |  | NR | NR | 45.5 (57.9) | 43.9 (55.6) | Morphine equivalent, mg  2-26 hour |
| **Li, 2020**^20^ | Tropisetron  243/251 (97) | NR | NR | IV patient administration of granisetron | NR | NR | NR | NR | Patient controlled analgesia device with IV administration of sufentanil |

*SD: standard deviation, NR: not reported, IV: intravenous, IM: intramuscular*

| Supplemental Table 4. Outcome - fraction of inspired oxygen | | | | | | | | | | | | | | | |
| --- | --- | --- | --- | --- | --- | --- | --- | --- | --- | --- | --- | --- | --- | --- | --- |
| **Trial** | **Pain** | | | **Shoulder pain** | | | **Nausea** | | | **Vomiting** | | | **PONV** | | |
|  | **E** | **L** | **T** | **E** | **L** | **T** | **E** | **L** | **T** | **E** | **L** | **T** | **E** | **L** | **T** |
| **Kurz, 2015**^3^ | X | X |  |  |  |  | X | X |  | X | X |  |  |  |  |
| **McKeen, 2015**^4^ |  |  |  |  |  |  | X | X |  | X | X |  | X | X | X |
| **Simurina, 2009**^5^ | X | X |  |  |  |  | X | X | X | X | X | X | X | X | X |
| **Goll, 2001**^6^ |  |  | X |  |  |  | X | X | X | X | X | X | X | X | X |
| **Joris, 2003**^7^ | X | X |  |  |  |  | X | X | X | X | X | X |  |  |  |
| **Mraovic, 2008**^8^ | X | X |  |  |  |  | X | X | X | X | X | X | X | X | X |
| **Purhonen, 2006**^9^ |  |  |  |  |  |  | X | X | X | X | X | X |  |  |  |
| **Purhonen, 2003**^10^ |  |  |  |  |  |  | X | X | X | X | X | X | X | X | X |
| **Thibon, 2012**^11^ |  |  |  |  |  |  |  |  |  |  |  |  |  |  | X |
| **Treschan, 2005**^12^ |  |  |  |  |  |  | X | X | X | X | X | X | X | X | X |
| **Purhonen, 2003**^13^ |  |  |  |  |  |  | X | X | X | X | X | X | X | X | X |
| **Sadrolsadat, 2007**^14^ |  |  |  |  |  |  |  |  |  |  |  |  |  |  | X |
| **Alvandipour, 2018**^15^ |  |  |  |  |  |  |  |  |  |  |  |  |  |  | X |
| **Meyhoff, 2009**^16^ |  |  |  |  |  |  |  |  |  |  |  |  |  |  | X |
| **Myles, 2007**^17^ |  |  |  |  |  |  |  |  |  |  |  |  |  |  | X |
| **Turan, 2006**^18^ |  |  |  |  |  |  | X | X | X | X | X | X | X | X | X |
| **Cohen, 2018**^19^ | X | X |  |  |  |  |  |  |  |  |  |  |  |  |  |
| **Li, 2020**^20^ |  |  |  |  |  |  |  |  |  |  |  |  |  |  | X |

*E: early, L: late, T: total, PONV: postoperative nausea and vomiting*

| Supplemental Table 5. Bias assessment - fraction of inspired oxygen | | | | | | |
| --- | --- | --- | --- | --- | --- | --- |
| **Trial** | **Randomization** | **Adherence to intervention** | **Missing outcome data** | **Measurement of the outcome** | **Selective reporting** | **Overall** |
| **Kurz, 2015**^3^ | Low | Intermediate | Low | Intermediate | Intermediate | Intermediate |
| **McKeen, 2015**^4^ | Low | Intermediate | Low | Low | Intermediate | Intermediate |
| **Simurina, 2009**^5^ | Low | Intermediate | Low | Low | Intermediate | Intermediate |
| **Goll, 2001**^6^ | Low | Intermediate | Low | Low | Intermediate | Intermediate |
| **Joris, 2003**^7^ | Low | Intermediate | Low | Low | Intermediate | Intermediate |
| **Mraovic, 2008**^8^ | Low | Intermediate | Low | Intermediate | Intermediate | Intermediate |
| **Purhonen, 2006**^9^ | Low | Intermediate | Low | Low | Intermediate | Intermediate |
| **Purhonen, 2003**^10^ | Low | Intermediate | Low | Low | Intermediate | Intermediate |
| **Thibon, 2012**^11^ | Low | Intermediate | Low | Low | Intermediate | Intermediate |
| **Treschan, 2005**^12^ | Low | Intermediate | Low | Intermediate | Intermediate | Intermediate |
| **Purhonen, 2003**^13^ | Low | Intermediate | Low | Low | Intermediate | Intermediate |
| **Sadrolsadat, 2007**^14^ | Low | Intermediate | Low | Low | Intermediate | Intermediate |
| **Alvandipour, 2018**^15^ | Low | Intermediate | Low | Low | Intermediate | Intermediate |
| **Meyhoff, 2009**^16^ | Low | Intermediate | Low | Low | Low | Intermediate |
| **Myles, 2007**^17^ | Low | Intermediate | Low | Low | Intermediate | Intermediate |
| **Turan, 2006**^18^ | Low | Intermediate | Low | Low | Intermediate | Intermediate |
| **Cohen, 2018**^19^ | Low | Intermediate | Low | Intermediate | Intermediate | Intermediate |
| **Li, 2020**^20^ | Low | Intermediate | Low | Low | Low | Intermediate |

| Supplemental Table 6: Results from meta-analyses | | | | | | |
| --- | --- | --- | --- | --- | --- | --- |
| **Outcome** | **Time** | **Number of trials (references)** | **Number of patients**  **Events/total or total^a^** | | **Heterogeneity (I^2^ [%])^b^** | **Effect estimate (95% CI)^c^** |
|  |  |  | **Intervention** | **Control** |  |  |
| ***Analysis: Fraction of inspired oxygen*** | | | | | | |
| **Nausea** | 0-6 hours | 9^3–7 9 10 12 18^ | 302/1,023 | 311/1,008 | 16 | 0.92 (0.73; 1.16) |
|  | 2-24 hours | 9^3–7 9 10 12 18^ | 312/1,023 | 312/1,008 | 24 | 0.94 (0.73; 1.21) |
|  | Total | 7^5–7 9 10 12 18^ | 210/591 | 217/594 | 47 | 0.84 (0.57; 1.23) |
| **Vomiting** | 0-6 hours | 9^3–7 9 10 12 18^ | 85/1,023 | 101/1,008 | 41 | 0.70 (0.41; 1.19) |
|  | 2-24 hours | 9^3–7 9 10 12 18^ | 124/1,023 | 140/1,008 | 0 | 0.85 (0.65; 1.11) |
|  | Total | 7^5–7 9 10 12 18^ | 98/591 | 124/594 | 0 | 0.74 (0.54; 1.01) |
| **PONV** | 0-6 hours | 6^4–6 10 12 18^ | 175/659 | 184/660 | 44 | 0.91 (0.63; 1.33) |
|  | 2-24 hours | 6^4–6 10 12 18^ | 170/659 | 163/660 | 37 | 0.98 (0.68; 1.41) |
| **Pain** | 0-2 and 0-6 hours | 4^3 5 7 19^ | 2,786 | 2,643 | 0 | -0.02 (-0.08; 0.03) |
|  | 2-24 and 6-24 hours | 4^3 5 7 19^ | 2,786 | 2,643 | 65 | -0.09 (-0.26; 0.09) |

*PONV: postoperatively nausea and vomiting, CI: confidence interval.*

^a^ Number of patients with events/total for nausea, vomiting and PONV and total number of patients for pain as outcome.

^b^ The I squared statistic (I^2^) is an estimation of the percentage of the variability in the effect estimates that is due to non-random variation.

^c^ Odds ratio for binary outcomes and mean differences (cm NRS) for pain. Effect estimates below 1 for odds ratios and below 0 for mean differences indicates better outcomes in the intervention group.

| Supplemental Table 7. GRADE - fraction of inspired oxygen | | | | | | | | | | | |
| --- | --- | --- | --- | --- | --- | --- | --- | --- | --- | --- | --- |
| **Certainty assessment** | | | | | | | **No of patients** | | **Effect** | | **Certainty** |
| **No of trials** | **Study design** | **Risk of bias** | **Inconsistency** | **Indirectness** | **Imprecision** | **Other** | **FiO_2_ 80%** | **FiO_2_ 30%** | **Relative (95% CI)** | **Absolute (95% CI)** |  |
| **Post-operatively nausea and vomiting** | | | | | | | | | | | |
| 12 | RCT | Serious^a^ | Serious^b^ | Not serious | Serious^c^ | None | 552/2783 (19.8%) | 693/2800 (24.8%) | **OR 0.75** (0.52 to 1.07) | **50 fewer per 1,000** (from 101 fewer to 13 more) | ⨁ ◯ ◯ ◯ VERY LOW |
| **Early pain** | | | | | | | | | | | |
| 4 | RCT | Serious^a^ | Not serious | Not serious | Not serious^c^ | None | 2786 | 2643 |  | **MD 0.02 lower**  (0.08 lower to 0.03 higher) | ⨁ ⨁⨁ ◯ MODERATE |
| **Late pain** | | | | | | | | | | | |
| 4 | RCT | Serious^a^ | Serious^d^ | Not serious | Not serious^c^ | None | 2786 | 2643 |  | **MD 0.09 lower**  (0.26 lower to 0.09 higher) | ⨁ ⨁ ◯ ◯  LOW |

*CI: confidence interval, OR: odds ratio, MD: mean difference, RCT: randomized clinical trial*

^a^ All trials were assessed as having an intermediate risk of bias

^b^ I^2^ = 81%, partly explained by surgery type

^c^ 95% confidence interval includes both benefit and no difference

^d^ I^2^ = 65%

# End-tidal or arterial carbon dioxide level

| Supplemental Table 8. Patient and surgical characteristics - end-tidal or arterial carbon dioxide level | | | | | | | | | | | | | | | | | |
| --- | --- | --- | --- | --- | --- | --- | --- | --- | --- | --- | --- | --- | --- | --- | --- | --- | --- |
| **Trial** | **n** | **Patient characteristics** | | | | | | | **Surgical characteristics** | | | | | | | | |
|  |  | **Age** | **Sex**  **(% male)** | **BMI** | **ASA** | | | | **Surgery** | | | | | **Laparoscopic (%)** | **Acute (%)** | **Duration (min.)** | |
|  |  |  |  |  | **1** | **2** | **3** | **4** | **Ort.** | **Abd.** | **Gyn.** | **Uro.** | **Oth.** |  |  | **Anesthesia** | **Surgery** |
| **Wang, 2019**^21^ | 90 | 61 | 59 | 25 | 0 | 64 | 36 | 0 | 0 | 100 | 0 | 0 | 0 | 100 | 0 | NR | 138 |
| **Mäkinen, 2000**^22^ | 24 | 49 | 33 | 26 | 63 | 37 | 0 | 0 | 0 | 100 | 0 | 0 | 0 | 100 | 0 | NR | NR |
| **Murphy, 2014**^23^ | 70 | 53 | 71 | 29 | NR | NR | NR | 0 | 100 | 0 | 0 | 0 | 0 | 0 | 0 | 97 | NR |
| **Saghaei, 2014**^24^ | 78 | 44 | 69 | 25 | NR | NR | NR | NR | 0 | 0 | 0 | 100 | 0 | 100 | NR | NR | 123 |
| **Akça, 2013**^25^ | 1215 | 52 | 51 | 27 | 23 | 58 | 19 | 0 | 0 | 100 | 0 | 0 | 0 | 29 | 0 | NR | NR |
| **Son, 2017**^26^ | 397 | 43 | 0 | 24 | NR | NR | 0 | 0 | 0 | 0 | 100 | 0 | 0 | 100 | NR | 106 | NR |
| **Besir, 2020**^27^ | 60 | 29 | 0 | 25 | 55 | 45 | 0 | 0 | 0 | 0 | 100 | 0 | 0 | 100 | 0 | NR | 66 |

*n: sample size, BMI: body mass index, ASA: American Society of Anesthesiologists, Ort: orthopedic, Abd.: abdominal, Gyn.: gynecological, Uro.: urological, Oth.: other, NR: not reported*

| Supplemental Table 9. Antiemetic and analgesia treatment - end-tidal or arterial carbon dioxide level | | | | | | | | | |
| --- | --- | --- | --- | --- | --- | --- | --- | --- | --- |
| **Trial** | **Prophylactic antiemetic treatment**  **n/total (%)** | **Any rescue antiemetic treatment**  **n/total (%)** | | | **Epidural treatment**  **n/ total (%)** | | **PACU opioid analgesia use**  **mean (SD)** | | |
|  |  | **Comparator** | **Intervention** | **Note** | **Comparator** | **Intervention** | **Comparator** | **Intervention** | **Note** |
| **Wang, 2019**^21^ | NR | NR | NR |  | NR | NR | NR | NR |  |
| **Mäkinen, 2000**^22^ | NR | NR | NR | Droperidol | NR | NR | 10.8 (5.4) | 13.3 (6.5) | Oxycodone, mg |
| **Murphy, 2014**^23^ | Ondansetron  70/70 (100) | NR | NR |  | NR | NR | 1.6 (2.3) | 2.0 (3.1) | Hydromorphone, mg |
| **Saghaei, 2014**^24^ | NR | NR | NR |  | NR | NR | 8.6 (1.5) | 7.7 (1.3)/ 7.8 (1.5) | Morphine, mg |
| **Akça, 2013**^25^ | NR | NR | NR |  | 80/616 (13) | 78/590 (13) | NR | NR | Patient controlled analgesia device with IV administration of morphine or hydromorphone |
| **Son, 2017**^26^ | Dexamethasone  381/381 (100) | 49/120 (41) | 48/134 (36)/  43/127 (34) | Palonosetron (1) and metoclopramide (2) | NR | NR | 673 (228) | 667 (240)/ 645 (204) | Patient controlled analgesia device with IV administration of fentanyl, µg |
| **Besir, 2020**^27^ | Ondansetron  60/60 (100) | 0/30 | 3/30 | Metoclopramide | NR | NR | NR | NR |  |

*SD: standard deviation, NR: not reported, IV: intravenous*

| Supplemental Table 10. Outcome - end tidal or arterial carbon dioxide level | | | | | | | | | | | | | | | |
| --- | --- | --- | --- | --- | --- | --- | --- | --- | --- | --- | --- | --- | --- | --- | --- |
| **Trial** | **Pain** | | | **Shoulder pain** | | | **Nausea** | | | **Vomiting** | | | **PONV** | | |
|  | **E** | **L** | **T** | **E** | **L** | **T** | **E** | **L** | **T** | **E** | **L** | **T** | **E** | **L** | **T** |
| **Wang, 2019**^21^ |  |  |  |  |  |  |  |  |  |  |  | (X) |  |  |  |
| **Mäkinen, 2000**^22^ |  |  |  |  |  |  |  |  | X |  |  |  |  |  |  |
| **Murphy, 2014**^23^ |  |  |  |  |  |  |  |  | X |  |  | X |  |  |  |
| **Saghaei, 2014**^24^ |  |  |  |  |  |  |  |  | X |  |  | X |  |  |  |
| **Akça, 2013**^25^ | X |  | X |  |  |  | X |  | X | X |  | X |  |  |  |
| **Son, 2017**^26^ | X | X | X |  |  |  | X | X | X | X | X | X |  |  |  |
| **Besir, 2020**^27^ |  |  |  |  |  |  | X | X |  | X | X |  | X | X |  |

*E: Early, L: late, T: total, PONV: postoperative nausea and vomiting, (X): no events*

| Supplemental Table 11. Bias assessment - end-tidal or arterial carbon dioxide level | | | | | | |
| --- | --- | --- | --- | --- | --- | --- |
| **Trial** | **Randomization** | **Adherence to intervention** | **Missing outcome data** | **Measurement of the outcome** | **Selective reporting** | **Overall** |
| **Wang, 2019**^21^ | Low | Intermediate | Low | Intermediate | Intermediate | Intermediate |
| **Mäkinen, 2000**^22^ | Low | Intermediate | Low | Intermediate | Intermediate | Intermediate |
| **Murphy, 2014**^23^ | Low | Intermediate | Low | Low | Low | Intermediate |
| **Saghaei, 2014**^24^ | Low | Intermediate | Low | Low | Low | Intermediate |
| **Akça, 2013**^25^ | Low | Intermediate | Low | Low | Low | Intermediate |
| **Son, 2017**^26^ | Low | Intermediate | Low | Low | Low | Intermediate |
| **Besir, 2020**^27^ | Low | Intermediate | Low | Intermediate | Low | Intermediate |

# Tidal volume and positive end-expiratory pressure

| Supplemental Table 12. Patient and surgical characteristics - tidal volume and positive end-expiratory pressure | | | | | | | | | | | | | | | | | |
| --- | --- | --- | --- | --- | --- | --- | --- | --- | --- | --- | --- | --- | --- | --- | --- | --- | --- |
| **Trial** | **n** | **Patient characteristics** | | | | | | | **Surgical characteristics** | | | | | | | | |
|  |  | **Age** | **Sex**  **(% male)** | **BMI** | **ASA** | | | | **Surgery** | | | | | **Laparoscopic (%)** | **Acute (%)** | **Duration (min.)** | |
|  |  |  |  |  | **1** | **2** | **3** | **4** | **Ort.** | **Abd.** | **Gyn.** | **Uro.** | **Oth.** |  |  | **Anesthesia** | **Surgery** |
| **Asida, 2015**^28^ | 104 | 41 | NR | 26 | 81 | 19 | 0 | 0 | 0 | 0 | 0 | 100 | 0 | 0 | 0 | NR | 136 |
| **Severgnini, 2013**^29^ | 56 | 66 | 62 | 25 | 18 | 73 | 9 | 0 | 0 | 100 | 0 | 0 | 0 | 0 | 0 | 208 | NR |
| **Haliloglu, 2017**^30^ | 50 | 62 | 100 | 28 | NR | NR | 0 | 0 | 0 | 0 | 0 | 100 | 0 | 100 | 0 | NR | 205 |
| **Mølsted, 2020**^31^ | 24 | 65 | 100 | 27 | NR | NR | NR | NR | 0 | 0 | 0 | 100 | 0 | 100 | 0 | 210 | 166 |
| **Shin, 2010**^32^ | 64 | 31 | 32 | 22 | 88 | 12 | 0 | 0 | 0 | 100 | 0 | 0 | 0 | 100 | 100 | 66 | 44 |
| **Soh, 2018**^33^ | 78 | 73 | 37 | 25 | NR | NR | NR | NR | 0 | 0 | 0 | 0 | 100 | 0 | NR | 210 | NR |
| **Bluth, 2019**^34^ | 1976 | 49 | 30 | 44 | 2 | 48 | 50 | 1 | 3 | 79 | 7 | 2 | 9 | 75 | 0 | 192 | 150 |
| **Wetterslev, 2001**^35^ | 40 | 59 | NR | 25 | NR | NR | 0 | 0 | 0 | 100 | 0 | 0 | 0 | 0 | 0 | 199 | NR |
| **Seo, 2018**^36^ | 42 | 49 | 46 | 24 | NR | NR | 0 | 0 | 100 | 0 | 0 | 0 | 0 | 0 | 0 | 255 | 201 |

*n: sample size, BMI: body mass index, ASA: American Society of Anesthesiologists, Ort: orthopedic, Abd.: abdominal, Gyn.: gynecological, Uro.: urological, Oth.: other, NR: not reported*

| Supplemental Table 13. Antiemetic and analgesia treatment - tidal volume and positive end-expiratory pressure | | | | | | | | | |
| --- | --- | --- | --- | --- | --- | --- | --- | --- | --- |
| **Trial** | **Prophylactic antiemetic treatment**  **n/total (%)** | **Any rescue antiemetic treatment**  **n/total (%)** | | | **Epidural treatment**  **n/ total (%)** | | **PACU opioid analgesia use**  **mean (SD)** | | |
|  |  | **Comparator** | **Intervention** | **Note** | **Comparator** | **Intervention** | **Comparator** | **Intervention** | **Note** |
| **Asida, 2015**^28^ | NR | NR | NR |  | 18/52 (35) | 16/52 (31) | 390.6 (65) | 372.1 (72) | Fentanyl, µg |
| **Severgnini, 2013**^29^ | NR | NR | NR |  | 19/27 (70) | 19/28 (68) | NR | NR |  |
| **Haliloglu, 2017**^30^ | NR | NR | NR |  | NR | NR | 20.4 (2.4) | 20.4 (1.8) | Patient controlled IV morphine, mg |
| **Mølsted, 2020**^31^ | Ondansetron  48/48 (100) | NR | NR |  | NR | NR | 3.2 (4.2)/ 10 (0) | 5 (0)/10 (0) | Morphine IV/ OR |
| **Shin, 2010**^32^ | NR | NR | NR |  | NR | NR | NR | NR |  |
| **Soh, 2018**^33^ | NR | NR | NR |  | NR | NR | NR | NR | Patient controlled IV fentanyl |
| **Bluth, 2019**^34^ | NR | NR | NR |  | 81/987 (8) | 80/989 (8) | NR | NR |  |
| **Wetterslev, 2001**^35^ | NR | NR | NR |  | 19/19 (100) | 19/19 (100) | 15.3 (27.1) | 14.2 (19.0) | Morphine IM |
| **Seo, 2018**^36^ | 0 | 0 | 0 |  | NR | NR | NR | NR |  |

*SD: standard deviation, NR: not reported, IV: intravenous, IM: intramuscular*

| Supplemental Table 14. Outcome - tidal volume and positive end-expiratory pressure | | | | | | | | | | | | | | | |
| --- | --- | --- | --- | --- | --- | --- | --- | --- | --- | --- | --- | --- | --- | --- | --- |
| **Trial** | **Pain** | | | **Shoulder pain** | | | **Nausea** | | | **Nemesis** | | | **PONV** | | |
|  | **E** | **L** | **La** | **E** | **L** | **La** | **E** | **L** | **La** | **E** | **L** | **La** | **E** | **L** | **La** |
| **Asida, 2015**^28^ |  |  | X |  |  |  |  |  |  |  |  |  |  |  |  |
| **Severgnini, 2013**^29^ | (X) | X | X |  |  |  |  |  |  |  |  |  |  |  |  |
| **Haliloglu, 2017**^30^ | X | X |  |  |  |  |  |  |  |  |  |  |  |  |  |
| **Mølsted, 2020**^31^ |  |  |  |  |  |  |  |  |  |  |  |  |  |  |  |
| **Shin, 2010**^32^ | X | X | X | X | X | X |  |  |  |  |  |  |  |  |  |
| **Soh, 2018**^33^ |  | X | X |  |  |  |  |  |  |  |  |  |  |  |  |
| **Bluth, 2019**^34^ |  | X | X |  | X | X |  |  |  |  |  |  |  |  |  |
| **Wetterslev, 2001**^35^ |  | X | X |  |  |  |  |  |  |  |  |  |  |  |  |
| **Seo, 2018**^36^ |  |  |  |  |  |  |  |  |  |  |  |  |  | (X) |  |

*E: Early, L: late, La: later, PONV: postoperative nausea and vomiting, (X): no events*

| Supplemental Table 15. Bias assessment - tidal volume and positive end-expiratory pressure | | | | | | |
| --- | --- | --- | --- | --- | --- | --- |
| **Trial** | **Randomization** | **Adherence to intervention** | **Missing outcome data** | **Measurement of the outcome** | **Selective reporting** | **Overall** |
| **Asida, 2015**^28^ | Low | Intermediate | Low | Intermediate | Intermediate | Intermediate |
| **Severgnini, 2013**^29^ | Low | Intermediate | Low | Intermediate | Low | Intermediate |
| **Haliloglu, 2017**^30^ | Low | Intermediate | Low | Intermediate | Intermediate | Intermediate |
| **Mølsted, 2020**^31^ | Low | Intermediate | Low | Low | Low | Intermediate |
| **Shin, 2010**^32^ | Low | Intermediate | Low | Low | Intermediate | Intermediate |
| **Soh, 2018**^33^ | Low | Intermediate | Low | Low | Low | Intermediate |
| **Bluth, 2019**^34^ | Low | Intermediate | Low | Intermediate | Low | Intermediate |
| **Wetterslev, 2001**^35^ | Low | Intermediate | Low | Intermediate | Intermediate | Intermediate |
| **Seo, 2018**^36^ | Low | Intermediate | Low | Intermediate | Low | Intermediate |

# Recruitment manoeuvre

| Supplemental Table 16. Patient and surgical characteristics - recruitment maneuver | | | | | | | | | | | | | | | | | |
| --- | --- | --- | --- | --- | --- | --- | --- | --- | --- | --- | --- | --- | --- | --- | --- | --- | --- |
| **Trial** | **n** | **Patient characteristics** | | | | | | | **Surgical characteristics** | | | | | | | | |
|  |  | **Age** | **Sex**  **(% male)** | **BMI** | **ASA** | | | | **Surgery** | | | | | **Laparoscopic (%)** | **Acute (%)** | **Duration (min.)** | |
|  |  |  |  |  | **1** | **2** | **3** | **4** | **Ort.** | **Abd.** | **Gyn.** | **Uro.** | **Oth.** |  |  | **Anesthesia** | **Surgery** |
| **Cho, 2020**^37^ | 287 | 39 | 0 | 23 | NR | NR | NR | NR | 0 | 0 | 100 | 0 | 0 | 100 | 0 | NR | 66 |
| **Khanna, 2013**^38^ | 88 | 48 | 49 | 28 | 50 | 43 | 7 | 0 | 0 | 100 | 0 | 0 | 0 | 100 | 0 | NR | NR |
| **Ryu, 2017**^39^ | 90 | 40 | 0 | 23 | 75 | 25 | 0 | 0 | 0 | 0 | 100 | 0 | 0 | 100 | 0 | NR | 63 |
| **Sharami, 2010**^40^ | 146 | 28 | 0 | 26 | NR | NR | 0 | 0 | 0 | 0 | 100 | 0 | 0 | 100 | NR | NR | 30 |
| **Davari-Tanha, 2019**^41^ | 280 | 31 | 0 | NR | NR | NR | 0 | 0 | 0 | 0 | 100 | 0 | 0 | 100 | 0 | NR | 80 |
| **Lee, 2020**^42^ | 84 | 41 | 0 | 23 | 76 | 24 | 0 | 0 | 0 | 0 | 100 | 0 | 0 | 100 | 0 | NR | 95 |
| **Pasquier, 2018**^43^ | 200 | 46 | 19 | 38 | NR | NR | 0 | 0 | 0 | 100 | 0 | 0 | 0 | 100 | 0 | NR | 66 |
| **Phelps, 2008**^44^ | 116 | 34 | 0 | 26 | NR | NR | 0 | 0 | 0 | 0 | 100 | 0 | 0 | 100 | 0 | NR | 43 |
| **Ryu, 2019**^45^ | 144 | 40 | 0 | 23 | 71 | 29 | 0 | 0 | 0 | 0 | 100 | 0 | 0 | 100 | 0 | NR | 59 |
| **Yilmaz, 2020**^46^ | 72 | 36 | 0 | 30 | NR | NR | 0 | 0 | 0 | 0 | 100 | 0 | 0 | 100 | 0 | NR | 109 |
| **Tsai, 2013**^47^ | 100 | 39 | 0 | 22 | NR | NR | 0 | 0 | 0 | 0 | 100 | 0 | 0 | 100 | 0 | NR | 147 |

*n: sample size, BMI: body mass index, ASA: American Society of Anesthesiologists, Ort: orthopedic, Abd.: abdominal, Gyn.: gynecological, Uro.: urological, Oth.: other, NR: not reported*

| Supplemental Table 17. Antiemetic and analgesia treatment - recruitment maneuver | | | | | | | | | |
| --- | --- | --- | --- | --- | --- | --- | --- | --- | --- |
| **Trial** | **Prophylactic antiemetic treatment**  **n/total (%)** | **Any rescue antiemetic treatment**  **n/total (%)** | | | **Epidural treatment**  **n/ total (%)** | | **PACU opioid analgesia use**  **mean (SD)** | | |
|  |  | **Comparator** | **Intervention** | **Note** | **Comparator** | **Intervention** | **Comparator** | **Intervention** | **Note** |
| **Cho, 2020**^37^ | NR | NR | NR |  | NR | NR | NR | NR | Meperidine, PN |
| **Khanna, 2013**^38^ | NR | NR | NR |  | NR | NR | NR | NR | Tramadol, 50mg every 6hour |
| **Ryu, 2017**^39^ | NR | NR | NR |  | NR | NR | NR | NR |  |
| **Sharami, 2010**^40^ | NR | NR | NR |  | NR | NR | NR | NR | Meperidine, 50mg, PN |
| **Davari-Tanha, 2019**^41^ | NR | NR | NR |  | NR | NR | NR | NR |  |
| **Lee, 2020**^42^ | Ondansetron  84/84 (100) | 42/42 (100) | 42/42 (100) | Ondansetron was part of the patient controlled IV analgesia | NR | NR | NR | NR | Patient controlled IV sufentanil  Tramadol, 50mg, PN |
| **Pasquier, 2018**^43^ | Ondansetron  21/150 (14)  Betamethasone  150/150 (100)  Droperidol  145/150 (97) | NR | NR |  | NR | NR | 9.7 (7.6) | 5.7 (6.1) | Opioids, mg |
| **Phelps, 2008**^44^ | NR | NR | NR |  | NR | NR | 36.3 (4.8) | 40.9 (3.7) | Meperidine, mg |
| **Ryu, 2019**^45^ | NR | NR | NR |  | NR | NR | NR | NR | Patient controlled IV fentanyl  Meperidine, mg, PN |
| **Yilmaz, 2020**^46^ | NR | NR | NR |  | NR | NR | NR | NR |  |
| **Tsai, 2013**^47^ | NR | NR | NR |  | NR | NR | 115.2 (43.72) | 104.2 (43.19) | Meperidine, mg, PN |

*SD: standard deviation, NR: not reported, IV: intravenous*

| Supplemental Table 18. Outcome - recruitment maneuver | | | | | | | | | | | | | | | |
| --- | --- | --- | --- | --- | --- | --- | --- | --- | --- | --- | --- | --- | --- | --- | --- |
| **Trial** | **Pain** | | | **Shoulder pain** | | | **Nausea** | | | **Vomiting** | | | **PONV** | | |
|  | **E** | **L** | **La** | **E** | **L** | **La** | **E** | **L** | **T** | **E** | **L** | **T** | **E** | **L** | **T** |
| **Cho, 2020**^37^ |  |  |  | X | X |  |  |  |  |  |  |  |  |  |  |
| **Khanna, 2013**^38^ | (X) | X | (X) |  |  |  |  |  |  |  |  |  |  |  |  |
| **Ryu, 2017**^39^ |  | X | X |  | X | X |  |  |  |  |  |  |  |  |  |
| **Sharami, 2010**^40^ |  |  |  | X | X | X |  |  |  |  |  |  |  |  |  |
| **Davari-Tanha, 2019**^41^ | X | X |  | X | X |  |  |  |  |  |  |  |  |  | X |
| **Lee, 2020**^42^ |  | X | X |  | X | X |  |  |  |  |  |  |  |  | X |
| **Pasquier, 2018**^43^ | X | X | X |  |  |  | X |  |  | X |  |  |  |  |  |
| **Phelps, 2008**^44^ |  |  |  |  | X | (X) |  |  |  |  |  |  |  |  | X |
| **Ryu, 2019**^45^ |  | X | X |  | X | X |  |  |  |  |  |  |  |  | X |
| **Yilmaz, 2020**^46^ | X | X |  | X | X |  |  |  |  |  |  |  |  |  |  |
| **Tsai, 2013**^47^ |  | X | X |  | X | X |  |  |  |  |  |  |  |  | X |

*E: Early, L: late, La: later, T: total, PONV: postoperative nausea and vomiting, (X): no range/standard deviation available*

| Supplemental Table 19. Bias assessment - recruitment maneuver | | | | | | |
| --- | --- | --- | --- | --- | --- | --- |
| **Trial** | **Randomization** | **Adherence to intervention** | **Missing outcome data** | **Measurement of the outcome** | **Selective reporting** | **Overall** |
| **Cho, 2020**^37^ | Low | Intermediate | Low | Low | Low | Intermediate |
| **Khanna, 2013**^38^ | Low | Low | Low | Low | Intermediate | Intermediate |
| **Ryu, 2017**^39^ | Low | Intermediate | Low | Low | Low | Intermediate |
| **Sharami, 2010**^40^ | Low | Intermediate | Low | Low | Intermediate | Intermediate |
| **Davari-Tanha, 2019**^41^ | Low | Intermediate | Low | Low | Low | Intermediate |
| **Lee, 2020**^42^ | Low | Intermediate | Low | Low | Low | Intermediate |
| **Pasquier, 2018**^43^ | Low | Intermediate | Intermediate | Low | Low | Intermediate |
| **Phelps, 2008**^44^ | Low | Intermediate | Low | Low | Low | Intermediate |
| **Ryu, 2019**^45^ | Low | Intermediate | Low | Intermediate | Low | Intermediate |
| **Yilmaz, 2020**^46^ | Low | Intermediate | Low | Low | Low | Intermediate |
| **Tsai, 2013**^47^ | Low | Intermediate | Low | Low | Low | Intermediate |

### Supplemental Figure 2: Recruitment maneuver and pain 48 hours postoperatively

Results from random-effects meta-analyses of trials assessing recruitment maneuvers. Results are displayed as mean differences (dots) with 95% confidence intervals (error bars). Values below 0 indicate reduced pain 48 hour postoperatively with recruitment maneuvers.

### Supplemental Figure 3: Recruitment maneuver and shoulder pain 48 hours postoperatively

Results from random-effects meta-analyses of trials assessing recruitment maneuvers. Results are displayed as mean differences (dots) with 95% confidence intervals (error bars). Values below 0 indicate reduced shoulder pain 48 hour postoperatively with recruitment maneuvers.

### Supplemental Figure 4: Recruitment maneuver and shoulder pain 4-6 hours postoperatively

Results from random-effects meta-analyses of trials assessing recruitment maneuvers. Results are displayed as mean differences (dots) with 95% confidence intervals (error bars). Values below 0 indicate reduced shoulder pain 4-6 hours postoperatively with recruitment maneuvers.

### Supplemental Figure 5: Recruitment maneuver and postoperatively nausea and vomiting

Results from random-effects meta-analyses of trials assessing recruitment maneuver. Results are displayed as odds ratios (dots) with 95% confidence intervals (error bars). Values below 1 indicate reduced postoperative nausea and vomiting with recruitment maneuver.

| Supplemental Table 20. GRADE - recruitment maneuver | | | | | | | | | | | |
| --- | --- | --- | --- | --- | --- | --- | --- | --- | --- | --- | --- |
| **Certainty assessment** | | | | | | | **No of patients** | | **Effect** | | **Certainty** |
| **No of trials** | **Study design** | **Risk of bias** | **Inconsistency** | **Indirectness** | **Imprecision** | **Other** | **Recruitment** | **No recruitment** | **Relative (95% CI)** | **Absolute (95% CI)** |  |
| **Pain** | | | | | | | | | | | |
| 7 | RCT | Serious^a^ | Serious^b^ | Not serious | Serious^c^ | None | 385 | 350 |  | MD -0.16  (0.49 lower to 0.18 higher) | ⨁ ◯ ◯ ◯ VERY LOW |
| **Shoulder pain** | | | | | | | | | | | |
| 7 |  | Serious^a^ | Serious^d^ | Not serious | Not serious^c^ | None | 453 | 411 |  | MD -1.10  (1.71 lower to 0.48 lower) | ⨁ ⨁ ◯ ◯ LOW |
| **Post-operatively nausea and vomiting** | | | | | | | | | | | |
| 6 |  | Serious^a^ | Serious^e^ | Not serious | Serious^f^ | None | 76/328 (23.2%) | 113/329 (34.3%) | OR 0.72 (0.31 to 1.71) | **70 fewer per 1,000**  (from 204 fewer to 129 more) | ⨁ ◯ ◯ ◯ VERY LOW |

*CI: confidence interval, OR: odds ratio, MD: mean difference, RCT: randomized clinical trial*

^a^ All trials were assessed as having an intermediate risk of bias

^b^ I^2^ = 92%

^c^ 95% confidence interval includes a small beneficial effect and no effect

^d^ I^2^ = 94%, primarily driven by one trial

^e^ I^2^ = 80%

^f^ 95% confidence interval includes both benefit and harm

# Goal-directed hemodynamic therapy

| Supplemental Table 21. Patient and surgical characteristics - goal-directed hemodynamic therapy | | | | | | | | | | | | | | | | | |
| --- | --- | --- | --- | --- | --- | --- | --- | --- | --- | --- | --- | --- | --- | --- | --- | --- | --- |
| **Trial** | **n** | **Patient characteristics** | | | | | | | **Surgical characteristics** | | | | | | | | |
|  |  | **Age** | **Sex**  **(% male)** | **BMI** | **ASA** | | | | **Surgery** | | | | | **Laparoscopic (%)** | **Acute (%)** | **Duration (min.)** | |
|  |  |  |  |  | **1** | **2** | **3** | **4** | **Ort.** | **Abd.** | **Gyn.** | **Uro.** | **Oth.** |  |  | **Anesthesia** | **Surgery** |
| **Bundgaard-Nielsen, 2013**^48^ | 44 | 64 | 100 | 26 | 62 | 38 | 0 | 0 | 0 | 0 | 0 | 100 | 0 | 0 | 0 | 214 | 157 |
| **Ramsingh, 2013**^49^ | 46 | 59 | 29 | 30 | NR | NR | NR | NR | 0 | 47 | 45 | 8 | 0 | 0 | NR | NR | 282 |
| **Liang, 2017**^50^ | 60 | 72 | 100 | 25 | 0 | 72 | 28 | 0 | 0 | 0 | 0 | 100 | 0 | 0 | 0 | NR | 120 |
| **Liu, 2019**^51^ | 74 | 70 | 62 | 22 | 15 | 85 | 0 | 0 | 0 | 100 | 0 | 0 | 0 | 100 | 0 | 220 | 181 |
| **Luo, 2017**^52^ | 150 | 62 | 43 | 23 | 0 | 0 | 97 | 3 | 0 | 0 | 0 | 0 | 100 | 0 | 0 | NR | 275 |
| **Noblett, 2006**^53^ | 108 | 65 | NR | 26 | NR | NR | NR | NR | 0 | 100 | 0 | 0 | 0 | 25 | 0 | NR | 158 |
| **Zhang, 2018**^54^ | 83 | 65 | 67 | 23 | 53 | 47 | 0 | 0 | 0 | 0 | 0 | 0 | 100 | NR | 0 | NR | 121 |
| **Zheng, 2013**^55^ | 65 | 68 | 50 | 21 | 0 | 40 | 60 | 0 | 0 | 100 | 0 | 0 | 0 | 0 | 0 | NR | 158 |
| **Peng, 2014**^56^ | 80 | 54 | 43 | 23 | 42 | 54 | 4 | 0 | 100 | 0 | 0 | 0 | 0 | 0 | 0 | NR | 169 |
| **Gan, 2002**^57^ | 100 | 56 | 57 | NR | 11 | 68 | 21 | 0 | 0 | 31 | 32 | 37 | 0 | NR | 0 | NR | NR |
| **Phan, 2014**^58^ | 100 | 64 | 61 | NR | NR | NR | NR | 0 | 0 | 100 | 0 | 0 | 0 | 59 | 0 | NR | 233 |
| **Weinberg, 2017**^59^ | 52 | 65 | 56 | 28 | NR | NR | NR | NR | 0 | 100 | 0 | 0 | 0 | 0 | 0 | NR | 492 |
| **Weinberg, 2019**^60^ | 50 | 63 | 64 | 27 | NR | NR | 78 | 0 | 0 | 100 | 0 | 0 | 0 | 0 | 0 | NR | NR |
| **Zhang, 2012**^61^ | 60 | 54 | 70 | 22 | 62 | 38 | 0 | 0 | 0 | 100 | 0 | 0 | 0 | 100 | 0 | NR | 185 |
| **Mühlbacher, 2021**^62^ | 60 | 38 | 18 | 45 | 12 | 72 | 16 | 0 | 0 | 100 | 0 | 0 | 0 | 100 | 0 | 159 | 108 |
| **Pillai, 2011**^63^ | 66 | 67 | 29 | 26 | NR | NR | NR | NR | 0 | 0 | 0 | 100 | 0 | NR | 0 | NR | 325 |

*n: sample size, BMI: body mass index, ASA: American Society of Anesthesiologists, Ort: orthopedic, Abd.: abdominal, Gyn.: gynecological, Uro.: urological, Oth.: other, NR: not reported*

| Supplemental Table 22. Antiemetic and analgesia treatment - goal-directed hemodynamic therapy | | | | | | | | | |
| --- | --- | --- | --- | --- | --- | --- | --- | --- | --- |
| **Trial** | **Prophylactic antiemetic treatment**  **n/total (%)** | **Any rescue antiemetic treatment**  **n/total (%)** | | | **Epidural treatment**  **n/ total (%)** | | **PACU opioid analgesia use**  **mean (SD)** | | |
|  |  | **Comparator** | **Intervention** | **Note** | **Comparator** | **Intervention** | **Comparator** | **Intervention** | **Note** |
| **Bundgaard-Nielsen, 2013**^48^ | NR | NR | NR |  | 0/21 (0) | 0/21 (0) | NR | NR | Oxycodone, 40mg, daily  Oxycodone PN |
| **Ramsingh, 2013**^49^ | NR | NR | NR |  | 2/20 (10) | 3/18 (17) | 188.4 (149.2) | 145.7 (70.2) | Morphine IV, mg |
| **Liang, 2017**^50^ | NR | NR | NR |  | NR | NR | NR | NR |  |
| **Liu, 2019**^51^ | NR | NR | NR | Patient controlled IV palonosetron | NR | NR | NR | NR | Patient controlled IV sufentanil |
| **Luo, 2017**^52^ | NR | NR | NR |  | NR | NR | NR | NR |  |
| **Noblett, 2006**^53^ | NR | NR | NR |  | 33/52 (63) | 32/51 (63) | NR | NR | Epidural or patient controlled IV analgesia |
| **Zhang, 2018**^54^ | NR | NR | NR |  | NR | NR | NR | NR | Patient controlled IV administration of sufentanil |
| **Zheng, 2013**^55^ | NR | NR | NR |  | NR | NR | NR | NR |  |
| **Peng, 2014**^56^ | NR | NR | NR |  | 0/22 (0) | 0/23 (0) | NR | NR | Patient controlled analgesia |
| **Gan, 2002**^57^ | Droperidol  100/100 (100) | NR | NR | Ondansetron =>2 emetic episodes or on patients request | 8/50 (16) | 6/50 (12) | NR | NR | Patient controlled IV fentanyl |
| **Phan, 2014**^58^ | NR | NR | NR |  | 7/50 (14) | 6/50 (12) | NR | NR | Patient controlled opioid analgesia |
| **Weinberg, 2017**^59^ | Dexamethason  52/52 (100) | NR | NR |  | 8/26 (31) | 7/26 (27) | NR | NR | Patient controlled IV fentanyl and oral oxycodone  Tramadol PN  Epidural analgesia  Ketamin infusion |
| **Weinberg, 2019**^60^ | NR | NR | NR | 15 mg i.v. metoclopramide (x3/day postoperatively day 0-2) | NR | NR | NR | NR | Patient controlled IV fentanyl, fentanyl infusion (PACU), and tramadol PN (ward) |
| **Zhang, 2012**^61^ | Metoclopramide  60/60 (100) | NR | NR |  | NR | NR | 59.0 (32.0) | 57.1 (29.3) | Morphine, mg |
| **Mühlbacher, 2021**^62^ | NR | 17/30 (57) | 17/30 (57) | Not specified | NR | NR | 8 (6) | 9 (6) | Piritramid, mg |
| **Pillai, 2011**^63^ | NR | NR | NR |  | NR | NR | NR | NR |  |

*SD: standard deviation, NR: not reported, IV: intravenous, IM: intramuscular, PACU: post-anesthesia care unit*

| Supplemental Table 23. Outcome - goal-directed hemodynamic therapy | | | | | | | | | | | | | | | |
| --- | --- | --- | --- | --- | --- | --- | --- | --- | --- | --- | --- | --- | --- | --- | --- |
| **Trial** | **Pain** | | | **Shoulder pain** | | | **Nausea** | | | **Vomiting** | | | **PONV** | | |
|  | **E** | **L** | **T** | **E** | **L** | **T** | **E** | **L** | **T** | **E** | **L** | **T** | **E** | **L** | **T** |
| **Bundgaard-Nielsen, 2013**^48^ |  |  | X |  |  |  |  |  |  |  |  |  |  |  |  |
| **Ramsingh, 2013**^49^ |  |  |  |  |  |  |  |  |  |  |  |  |  |  |  |
| **Liang, 2017**^50^ |  |  |  |  |  |  |  |  |  |  |  |  |  |  | X |
| **Liu, 2019**^51^ |  |  |  |  |  |  |  |  |  |  |  |  |  |  | X |
| **Luo, 2017**^52^ |  |  |  |  |  |  |  |  |  |  |  | X |  |  |  |
| **Noblett, 2006**^53^ |  |  |  |  |  |  |  |  |  |  |  |  |  |  |  |
| **Zhang, 2018**^54^ |  |  |  |  |  |  |  |  |  |  |  |  |  |  | X |
| **Zheng, 2013**^55^ |  |  |  |  |  |  |  |  |  |  |  |  |  |  | X |
| **Peng, 2014**^56^ |  |  |  |  |  |  |  |  | X |  |  | X |  |  |  |
| **Gan, 2002**^57^ |  |  |  |  |  |  |  |  |  |  |  |  |  |  | X |
| **Phan, 2014**^58^ |  |  | (X) |  |  |  |  |  |  |  |  |  |  |  | X |
| **Weinberg, 2017**^59^ |  |  |  |  |  |  |  |  |  |  |  |  |  |  | X |
| **Weinberg, 2019**^60^ |  |  |  |  |  |  |  |  |  |  |  |  |  |  | X |
| **Zhang, 2012**^61^ |  |  |  |  |  |  |  |  |  |  |  |  |  |  | X |
| **Mühlbacher, 2021**^62^ |  |  | X |  |  |  |  |  |  |  |  |  |  |  | X |
| **Pillai, 2011**^63^ |  |  |  |  |  |  |  |  |  |  |  |  | X | X | X |

*E: early, L: late, T: total, PONV: postoperative nausea and vomiting, (X): only numbers available*

| Supplemental Table 24. Bias assessment - goal-directed hemodynamic therapy | | | | | | |
| --- | --- | --- | --- | --- | --- | --- |
| **Trial** | **Randomization** | **Adherence to intervention** | **Missing outcome data** | **Measurement of the outcome** | **Selective reporting** | **Overall** |
| **Bundgaard-Nielsen, 2013**^48^ | Low | Intermediate | Low | Low | Low | Intermediate |
| **Ramsingh, 2013**^49^ | Low | Intermediate | Intermediate | Low | Intermediate | Intermediate |
| **Liang, 2017**^50^ | Low | Intermediate | Low | Intermediate | Intermediate | Intermediate |
| **Liu, 2019**^51^ | Low | Intermediate | Low | Low | Low | Intermediate |
| **Luo, 2017**^52^ | Low | Intermediate | Low | Intermediate | Intermediate | Intermediate |
| **Noblett, 2006**^53^ | Low | Low | Low | Low | Intermediate | Intermediate |
| **Zhang, 2018**^54^ | Intermediate | Intermediate | Low | Low | Low | Intermediate |
| **Zheng, 2013**^55^ | Low | Intermediate | Low | Intermediate | Intermediate | Intermediate |
| **Peng, 2014**^56^ | Low | Intermediate | Low | Low | Intermediate | Intermediate |
| **Gan, 2002**^57^ | Low | Intermediate | Low | Intermediate | Intermediate | Intermediate |
| **Phan, 2014**^58^ | Low | Intermediate | Low | Low | Low | Intermediate |
| **Weinberg, 2017**^59^ | Low | Intermediate | Low | Intermediate | Low | Intermediate |
| **Weinberg, 2019**^60^ | Low | Intermediate | Low | Low | Low | Intermediate |
| **Zhang, 2012**^61^ | Low | Intermediate | Low | Intermediate | Intermediate | Intermediate |
| **Mühlbacher, 2021**^62^ | Low | Intermediate | Low | Intermediate | Low | Intermediate |
| **Pillai, 2011**^63^ | Low | Low | Low | Low | Intermediate | Intermediate |

| Supplemental Table 25. GRADE - Goal-directed hemodynamic therapy | | | | | | | | | | | |
| --- | --- | --- | --- | --- | --- | --- | --- | --- | --- | --- | --- |
| **Certainty assessment** | | | | | | | **No of patients** | | **Effect** | | **Certainty** |
| **No of trials** | **Study design** | **Risk of bias** | **Inconsistency** | **Indirectness** | **Imprecision** | **Other** | **GDHT** | **No GDHT** | **Relative (95% CI)** | **Absolute (95% CI)** |  |
| **Post-operatively nausea and vomiting** | | | | | | | | | | | |
| 11 | RCT | Serious^a^ | Serious^b^ | Not serious | Serious^c^ | None | 62/379 (16.4%) | 110/361 (30.5%) | **OR 0.44** (0.24 to 0.78) | **143 fewer per 1,000**  (from 210 fewer to 50 fewer) | ⨁ ◯ ◯ ◯ VERY LOW |

*CI: confidence interval, OR: odds ratio, MD: mean difference, RCT: randomized clinical trial*

^a^ All trials were assessed as having an intermediate risk of bias

^b^ I^2^ = 44%

^c^ Optimal information size not reached

# Other interventions

| Supplemental Table 26. Patient and surgical characteristics - other interventions | | | | | | | | | | | | | | | | | |
| --- | --- | --- | --- | --- | --- | --- | --- | --- | --- | --- | --- | --- | --- | --- | --- | --- | --- |
| **Trial** | **n** | **Patient characteristics** | | | | | | | **Surgical characteristics** | | | | | | | | |
|  |  | **Age** | **Sex**  **(% male)** | **BMI** | **ASA** | | | | **Surgery** | | | | | **Laparoscopic (%)** | **Acute (%)** | **Duration (min.)** | |
|  |  |  |  |  | **1** | **2** | **3** | **4** | **Ort.** | **Abd.** | **Gyn.** | **Uro.** | **Oth.** |  |  | **Anesthesia** | **Surgery** |
| **Delfino, 2015**^64^ | 50 | 40 | 28 | 26 | 68 | 32 | 0 | 0 | 0 | 100 | 0 | 0 | 0 | 100 | 0 | 90 | 63 |
| **Xu, 2020**^65^ | 156 | 69 | 40 | 24 | 0 | 75 | 25 | 0 | 100 | 0 | 0 | 0 | 0 | NR | 0 | 152 | 115 |
| **Kim, 2019**^66^ | 84 | 48 | 25 | 23 | NR | NR | NR | 0 | 100 | 0 | 0 | 0 | 0 | 0 | 0 | 155 | NR |
| **Le Guen, 2019**^67^ | 86 | 49 | 47 | 26 | 17 | 77 | 4 | NR | 0 | 0 | 0 | 0 | 100 | 0 | 0 | NR | 41 |

*n: sample size, BMI: body mass index, ASA: American Society of Anesthesiologists, Ort: orthopedic, Abd.: abdominal, Gyn.: gynecological, Uro.: urological, Oth.: other, NR: not reported*

| Supplemental Table 27. Antiemetic and analgesia treatment - other interventions | | | | | | | | | |
| --- | --- | --- | --- | --- | --- | --- | --- | --- | --- |
| **Trial** | **Prophylactic antiemetic treatment**  **n/total (%)** | **Any rescue antiemetic treatment**  **n/total (%)** | | | **Epidural treatment**  **n/ total (%)** | | **PACU opioid analgesia use**  **mean (SD)** | | |
|  |  | **Comparator** | **Intervention** | **Note** | **Comparator** | **Intervention** | **Comparator** | **Intervention** | **Note** |
| **Delfino, 2015**^64^ | NR | NR | NR |  | NR | NR | 19 | 7 | Morphine, mg |
| **Xu, 2020**^65^ | NR | NR | NR | Granisetron was part of the patient controlled IV analgesia | NR | NR | NR | NR | Patient controlled IV sufentanil |
| **Kim, 2019**^66^ | NR | NR | NR |  | NR | NR | 101.7 (35.1) | 95.4 (42.7) | Fentanyl, μg |
| **Le Guen, 2019**^67^ | NR | NR | NR |  | NR | NR | NR | NR |  |

*SD: standard deviation, NR: not reported, IV: intravenous*

| Supplemental Table 28. Outcome - other interventions | | | | | | | | | | | | | | | |
| --- | --- | --- | --- | --- | --- | --- | --- | --- | --- | --- | --- | --- | --- | --- | --- |
| **Trial** | **Pain** | | | **Shoulder pain** | | | **Nausea** | | | **Vomiting** | | | **PONV** | | |
|  | **E** | **L** | **T** | **E** | **L** | **T** | **E** | **L** | **T** | **E** | **L** | **T** | **E** | **L** | **T** |
| **Delfino, 2015**^64^ | X | X |  |  |  |  |  |  |  |  |  |  |  |  | X |
| **Xu, 2020**^65^ | X | X |  |  |  |  |  |  |  |  |  |  |  |  |  |
| **Kim, 2019**^66^ | X |  |  |  |  |  |  |  |  |  |  |  |  |  |  |
| **Le Guen, 2019**^67^ | X |  |  |  |  |  |  |  |  |  |  |  |  |  |  |

*E: Early, L: late, T: total, PONV: postoperative nausea and vomiting*

| Supplemental Table 29. Bias assessment - other interventions | | | | | | |
| --- | --- | --- | --- | --- | --- | --- |
| **Trial** | **Randomization** | **Adherence to intervention** | **Missing outcome data** | **Measurement of the outcome** | **Selective reporting** | **Overall** |
| **Delfino, 2015**^64^ | Low | Intermediate | Low | Low | Low | Intermediate |
| **Xu, 2020**^65^ | Low | Intermediate | Low | Intermediate | Low | Intermediate |
| **Kim, 2019**^66^ | Low | Low | Low | Intermediate | Low | Low |
| **Le Guen, 2019**^67^ | Low | Intermediate | Low | Low | Low | Intermediate |

# PRISMA-checklist

| **Section and Topic** | **Item #** | **Checklist item** | **Location where item is reported** |
| --- | --- | --- | --- |
| **TITLE** | | |  |
| Title | 1 | Identify the report as a systematic review. | Page 1 |
| **ABSTRACT** | | |  |
| Abstract | 2 | See the PRISMA 2020 for Abstracts checklist. | Page 3 |
| **INTRODUCTION** | | |  |
| Rationale | 3 | Describe the rationale for the review in the context of existing knowledge. | Page 4 |
| Objectives | 4 | Provide an explicit statement of the objective(s) or question(s) the review addresses. | Page 4 |
| **METHODS** | | |  |
| Eligibility criteria | 5 | Specify the inclusion and exclusion criteria for the review and how studies were grouped for the syntheses. | Page 5 |
| Information sources | 6 | Specify all databases, registers, websites, organizations, reference lists and other sources searched or consulted to identify studies. Specify the date when each source was last searched or consulted. | Page 5-6 |
| Search strategy | 7 | Present the full search strategies for all databases, registers and websites, including any filters and limits used. | Page 5-6 |
| Selection process | 8 | Specify the methods used to decide whether a study met the inclusion criteria of the review, including how many reviewers screened each record and each report retrieved, whether they worked independently, and if applicable, details of automation tools used in the process. | Page 6 |
| Data collection process | 9 | Specify the methods used to collect data from reports, including how many reviewers collected data from each report, whether they worked independently, any processes for obtaining or confirming data from study investigators, and if applicable, details of automation tools used in the process. | Page 6 |
| Data items | 10a | List and define all outcomes for which data were sought. Specify whether all results that were compatible with each outcome domain in each study were sought (e.g. for all measures, time points, analyses), and if not, the methods used to decide which results to collect. | Page 6 |
|  | 10b | List and define all other variables for which data were sought (e.g. participant and intervention characteristics, funding sources). Describe any assumptions made about any missing or unclear information. | Page 6 |
| Study risk of bias assessment | 11 | Specify the methods used to assess risk of bias in the included studies, including details of the tool(s) used, how many reviewers assessed each study and whether they worked independently, and if applicable, details of automation tools used in the process. | Page 6  Supplementary |
| Effect measures | 12 | Specify for each outcome the effect measure(s) (e.g. risk ratio, mean difference) used in the synthesis or presentation of results. | Page 7 |
| Synthesis methods | 13a | Describe the processes used to decide which studies were eligible for each synthesis (e.g. tabulating the study intervention characteristics and comparing against the planned groups for each synthesis (item #5)). | Page 5-6 |
|  | 13b | Describe any methods required to prepare the data for presentation or synthesis, such as handling of missing summary statistics, or data conversions. | Page 7 |
|  | 13c | Describe any methods used to tabulate or visually display results of individual studies and syntheses. | Page 7 |
|  | 13d | Describe any methods used to synthesize results and provide a rationale for the choice(s). If meta-analysis was performed, describe the model(s), method(s) to identify the presence and extent of statistical heterogeneity, and software package(s) used. | Page 7 |
|  | 13e | Describe any methods used to explore possible causes of heterogeneity among study results (e.g. subgroup analysis, meta-regression). | Page 7 |
|  | 13f | Describe any sensitivity analyses conducted to assess robustness of the synthesized results. | Page 7 |
| Reporting bias assessment | 14 | Describe any methods used to assess risk of bias due to missing results in a synthesis (arising from reporting biases). | Page 6  Supplementary |
| Certainty assessment | 15 | Describe any methods used to assess certainty (or confidence) in the body of evidence for an outcome. | Page 7 |
| **RESULTS** | | |  |
| Study selection | 16a | Describe the results of the search and selection process, from the number of records identified in the search to the number of studies included in the review, ideally using a flow diagram. | Page 8  Supplementary |
|  | 16b | Cite studies that might appear to meet the inclusion criteria, but which were excluded, and explain why they were excluded. | Page 8  Supplementary |
| Study characteristics | 17 | Cite each included study and present its characteristics. | Page 8-11  Supplementary |
| Risk of bias in studies | 18 | Present assessments of risk of bias for each included study. | Supplementary |
| Results of individual studies | 19 | For all outcomes, present, for each study: (a) summary statistics for each group (where appropriate) and (b) an effect estimate and its precision (e.g. confidence/credible interval), ideally using structured tables or plots. | Page 8-11  Supplementary |
| Results of syntheses | 20a | For each synthesis, briefly summarize the characteristics and risk of bias among contributing studies. | Page 8-11  Supplementary |
|  | 20b | Present results of all statistical syntheses conducted. If meta-analysis was done, present for each the summary estimate and its precision (e.g. confidence/credible interval) and measures of statistical heterogeneity. If comparing groups, describe the direction of the effect. | Page 8-11  Supplementary |
|  | 20c | Present results of all investigations of possible causes of heterogeneity among study results. | Page 8-11  Supplementary |
|  | 20d | Present results of all sensitivity analyses conducted to assess the robustness of the synthesized results. | Page 8-11  Supplementary |
| Reporting biases | 21 | Present assessments of risk of bias due to missing results (arising from reporting biases) for each synthesis assessed. | Supplementary |
| Certainty of evidence | 22 | Present assessments of certainty (or confidence) in the body of evidence for each outcome assessed. | Page 8-11  Supplementary |
| **DISCUSSION** | | |  |
| Discussion | 23a | Provide a general interpretation of the results in the context of other evidence. | Page 12-13 |
|  | 23b | Discuss any limitations of the evidence included in the review. | Page 12-13 |
|  | 23c | Discuss any limitations of the review processes used. | Page 13 |
|  | 23d | Discuss implications of the results for practice, policy, and future research. | Page 12-13 |
| **OTHER INFORMATION** | | |  |
| Registration and protocol | 24a | Provide registration information for the review, including register name and registration number, or state that the review was not registered. | Page 5 |
|  | 24b | Indicate where the review protocol can be accessed, or state that a protocol was not prepared. | Page 5 |
|  | 24c | Describe and explain any amendments to information provided at registration or in the protocol. | Page 5 |
| Support | 25 | Describe sources of financial or non-financial support for the review, and the role of the funders or sponsors in the review. | Page 2 |
| Competing interests | 26 | Declare any competing interests of review authors. | Page 2 |
| Availability of data, code and other materials | 27 | Report which of the following are publicly available and where they can be found: template data collection forms; data extracted from included studies; data used for all analyses; analytic code; any other materials used in the review. | Supplementary |

# References

1. Higgins J, Sterne J, Savović J. A revised tool for assessing risk of bias in randomized trials. *Cochrane Database Syst Rev* 2016; Suppl 1

2. Guyatt GH, Oxman AD, Kunz R, et al. GRADE guidelines 6. Rating the quality of evidence - Imprecision. *J Clin Epidemiol* 2011; **64**: 1283–93

3. Kurz A, Fleischmann E, Sessler DI, Buggy DJ, Apfel C, Akcą O. Effects of supplemental oxygen and dexamethasone on surgical site infection: A factorial randomized trial. *Br J Anaesth* 2015; **115**: 434–43

4. McKeen DM, Arellano R, O’Connell C. Supplemental oxygen does not prevent postoperative nausea and vomiting after gynecological laparoscopy. *Can J Anesth* 2009; **56**: 651–7

5. Šimurina T, Mraović B, Mikulandra S, et al. Effects of high intraoperative inspired oxygen on postoperative nausea and vomiting in gynecologic laparoscopic surgery. *J Clin Anesth* Elsevier; 2010; **22**: 492–8

6. Goll V, Akça O, Greif R, et al. Ondansetron is no more effective than supplemental intraoperative oxygen for prevention of postoperative nausea and vomiting. *Anesth Analg* 2001; **92**: 112–7

7. Joris JL, Poth NJ, Djamadar AM, et al. Supplemental oxygen does not reduce postoperative nausea and vomiting after thyroidectomy. Br. J. Anaesth. 2003

8. Mraovic B, Šimurina T, Sonicki Z, Skitarelić N, Gan TJ. The dose-response of nitrous oxide in postoperative nausea in patients undergoing gynecologic laparoscopic surgery: A preliminary study. *Anesth Analg* 2008; **107**: 818–23

9. Purhonen S, Niskanen M, Wüstefeld M, Hirvonen E, Hynynen M. Supplemental 80% oxygen does not attenuate post-operative nausea and vomiting after breast surgery. *Acta Anaesthesiol Scand* John Wiley & Sons, Ltd; 2006; **50**: 26–31

10. Purhonen S, Turunen M, Ruohoaho U-M, Niskanen M, Hynynen M. Supplemental Oxygen Does Not Reduce the Incidence of Postoperative Nausea and Vomiting After Ambulatory Gynecologic Laparoscopy. *Anesth Analg* 2003; **96**: 91–6

11. Thibon P, Borgey F, Boutreux S, Hanouz JL, Le Coutour X, Parienti JJ. Effect of perioperative oxygen supplementation on 30-day surgical site infection rate in abdominal, gynecologic, and breast surgery: The ISO2 randomized controlled trial. Anesthesiology. 2012

12. Treschan TA, Zimmer C, Nass C, Stegen B, Esser J, Peters J. Inspired oxygen fraction of 0.8 does not attenuate postoperative nausea and vomiting after strabismus surgery. Anesthesiology. 2005

13. Purhonen S, Niskanen M, Wüstefeld M, Mustonen P, Hynynen M. Supplemental oxygen for prevention of nausea and vomiting after breast surgery. *Br J Anaesth* Elsevier; 2003; **91**: 284–7

14. Sadrolsadat SH, Shoroghi M, Farahbakhsh F, Moharreri RS, Sheikhvatan M, Abbasi A. The effect of supplemental 70% oxygen on postoperative nausea and vomiting in patients undergoing inguinal hernia surgery. *Hernia* Springer; 2008; **12**: 167–71

15. Alvandipour M, Mokhtari-Esbuie F, Baradari AG, Firouzian A, Rezaie M. Effect of hyperoxygenation during surgery on surgical site infection in colorectal surgery. *Ann Coloproctol* 2019; **35**: 9–14

16. Meyhoff CS, Wetterslev J, Jorgensen LN, et al. Effect of high perioperative oxygen fraction on surgical site infection and pulmonary complications after abdominal surgery: The PROXI randomized clinical trial. *JAMA - J Am Med Assoc* 2009; **302**: 1543–50

17. Myles PS, Leslie K, Chan MTV, et al. Avoidance of nitrous oxide for patients undergoing major surgery: A randomized controlled trial. Anesthesiology. 2007

18. Turan A, Apfel CC, Kumpch M, et al. Does the efficacy of supplemental oxygen for the prevention of postoperative nausea and vomiting depend on the measured outcome, observational period or site of surgery? *Anaesthesia* John Wiley & Sons, Ltd; 2006; **61**: 628–33

19. Cohen B, Ahuja S, Schacham YN, et al. Intraoperative Hyperoxia Does Not Reduce Postoperative Pain: Subanalysis of an Alternating Cohort Trial. *Anesth Analg* 2019; **128**: 1160–6

20. Li XF, Jiang D, Jiang YL, et al. Comparison of low and high inspiratory oxygen fraction added to lung-protective ventilation on postoperative pulmonary complications after abdominal surgery: A randomized controlled trial. *J Clin Anesth* 2020; **67**: 110009

21. Wang L, Yang L, Yang J, Shan S. Effects of Permissive Hypercapnia on Laparoscopic Surgery for Rectal Carcinoma. *Gastroenterol Res Pract* [Internet] Gastroenterol Res Pract; 2019 [cited 2021 Oct 12]; **2019**: 3903451 Available from: http://www.ncbi.nlm.nih.gov/pubmed/31687013

22. Mäkinen MT, Heinonen PO, Klemola UM, Yli-Hankala A. Gastric air tonometry during laparoscopic cholecystectomy: a comparison of two PaCO2 levels. *Can J Anaesth* [Internet] Can J Anaesth; 2001 [cited 2021 Oct 12]; **48**: 121–8 Available from: http://www.ncbi.nlm.nih.gov/pubmed/11220419

23. Murphy GS, Szokol JW, Avram MJ, et al. Effect of ventilation on cerebral oxygenation in patients undergoing surgery in the beach chair position: a randomized controlled trial. *Br J Anaesth* [Internet] Br J Anaesth; 2014 [cited 2021 Oct 12]; **113**: 618–27 Available from: http://www.ncbi.nlm.nih.gov/pubmed/24860157

24. Saghaei M, Matin G, Golparvar M. Effects of intra-operative end-tidal carbon dioxide levels on the rates of post-operative complications in adults undergoing general anesthesia for percutaneous nephrolithotomy: A clinical trial. *Adv Biomed Res* [Internet] 2014 [cited 2021 Oct 12]; **3**: 84 Available from: www.advbiores.net

25. Akça O, Kurz A, Fleischmann E, et al. Hypercapnia and surgical site infection: a randomized trial. *Br J Anaesth* [Internet] Elsevier; 2013 [cited 2021 Oct 12]; **111**: 759–67 Available from: http://www.ncbi.nlm.nih.gov/pubmed/23887247

26. Son J-S, Oh J-Y, Ko S. Effects of hypercapnia on postoperative nausea and vomiting after laparoscopic surgery: a double-blind randomized controlled study. *Surg Endosc* [Internet] Surg Endosc; 2017 [cited 2021 Oct 12]; **31**: 4576–82 Available from: http://www.ncbi.nlm.nih.gov/pubmed/28389799

27. Besir A, Tugcugil E. Comparison of different end-tidal carbon dioxide levels in preventing postoperative nausea and vomiting in gynaecological patients undergoing laparoscopic surgery. *J Obstet Gynaecol (Lahore)* 2021; **41**: 755–62

28. Asida SM, Badawy MS. Effect of low tidal volume during general anesthesia for urological procedures on lung fun ctions. *Egypt J Anaesth* 2015; **31**: 127–34

29. Severgnini P, Selmo G, Lanza C, et al. Protective mechanical ventilation during general anesthesia for open abdominal surgery improves postoperative pulmonary function. *Anesthesiology* Anesthesiology; 2013; **118**: 1307–21

30. Haliloglu M, Bilgili B, Ozdemir M, Umuroglu T, Bakan N. Low tidal volume positive end-expiratory pressure versus high tidal volume zero-positive end-expiratory pressure and postoperative pulmonary functions in robot-Assisted laparoscopic radical prostatectomy. *Med Princ Pract* Med Princ Pract; 2018; **26**: 573–8

31. Mølsted M, Ekeløf P, Bech JN, Wessels J, Jensen JB. Effects of lung protective ventilation on postoperative respiratory parameters in patients undergoing robot-assisted radical prostatectomy. *J Robot Surg* J Robot Surg; 2020; **14**: 509–16

32. Shin HY, Kim SH, Lee YJ, Kim DK. The effect of mechanical ventilation tidal volume during pneumoperitoneum on shoulder pain after a laparoscopic appendectomy. *Surg Endosc* Surg Endosc; 2010; **24**: 2002–7

33. Soh S, Shim JK, Ha Y, Kim YS, Lee H, Kwak YL. Ventilation with High or Low Tidal Volume with PEEP Does Not Influence Lung Function after Spinal Surgery in Prone Position: A Randomized Controlled Trial. *J Neurosurg Anesthesiol* J Neurosurg Anesthesiol; 2018; **30**: 237–45

34. Bluth T, Serpa Neto A, Schultz MJ, et al. Effect of Intraoperative High Positive End-Expiratory Pressure (PEEP) with Recruitment Maneuvers vs Low PEEP on Postoperative Pulmonary Complications in Obese Patients: A Randomized Clinical Trial. *JAMA - J Am Med Assoc* JAMA; 2019; **321**: 2292–305

35. Wetterslev J, Hansen EG, Roikjaer O, Kanstrup IL, Heslet L. Optimizing peroperative compliance with PEEP during upper abdominal surgery: Effects on perioperative oxygenation and complications in patients without preoperative cardiopulmonary dysfunction. *Eur J Anaesthesiol* Eur J Anaesthesiol; 2001; **18**: 358–65

36. Seo H, Do Son J, Lee HC, Oh HM, Jung CW, Park HP. Effects of positive end-expiratory pressure on intraoperative core temperature in patients undergoing posterior spine surgery: prospective randomised trial. *J Int Med Res* [Internet] J Int Med Res; 2018; **46**: 984–95 Available from: 243

37. Cho M, Kim CJ, Hahm TS, et al. Combination of a pulmonary recruitment maneuver and intraperitoneal bupivacaine for the reduction of postoperative shoulder pain in gynecologic laparoscopy: A randomized, controlled trial. *Obstet Gynecol Sci* Obstet Gynecol Sci; 2020; **63**: 187–94

38. Khanna A, Sezen E, Barlow A, Rayt H, Finch JG. Randomized clinical trial of a simple pulmonary recruitment manoeuvre to reduce pain after laparoscopy. *Br J Surg* Br J Surg; 2013; **100**: 1290–4

39. Ryu K, Choi W, Shim J, Song T. The impact of a pulmonary recruitment maneuver to reduce post-laparoscopic shoulder pain: A randomized controlled trial. *Eur J Obstet Gynecol Reprod Biol* Eur J Obstet Gynecol Reprod Biol; 2017; **208**: 55–60

40. Sharami SH, Sharami MB, Abdollahzadeh M, Keyvan A. Randomised clinical trial of the influence of pulmonary recruitment manoeuvre on reducing shoulder pain after laparoscopy. *J Obstet Gynaecol (Lahore)* J Obstet Gynaecol; 2010; **30**: 505–10

41. Davari-Tanha F, Samimi S, Khalaj Z, Bastanhagh E. Comparison of intraperitoneal normal saline infusion with pulmonary recruitment maneuver in reducing shoulder and upper abdomen pain following gynecologic laparoscopic procedures: A randomized, controlled, triple-blind trial. *Anesthesiol Pain Med* Kowsar; 2019; **9**

42. Lee J, Park C, Kim J, Ki Y, Cha SH, Kim JY. Effect of Low-pressure Pulmonary Recruitment Maneuver on Postlaparoscopic Shoulder Pain: Randomized Controlled Trial. *J Minim Invasive Gynecol* J Minim Invasive Gynecol; 2020; **27**: 173–7

43. Pasquier EK, Andersson E. Pulmonary recruitment maneuver reduces pain after laparoscopic bariatric surgery: a randomized controlled clinical trial. *Surg Obes Relat Dis* Surg Obes Relat Dis; 2018; **14**: 386–92

44. Phelps P, Cakmakkaya OS, Apfel CC, Radke OC. A simple clinical maneuver to reduce laparoscopy-induced shoulder pain: A randomized controlled trial. *Obstet Gynecol* Obstet Gynecol; 2008; **111**: 1155–60

45. Ryu KH, Lee SH, Cho EA, Kim JA, Lim GE, Song T. Comparison of impacts of intraperitoneal saline instillation with and without pulmonary recruitment maneuver on post-laparoscopic shoulder pain prevention: a randomized controlled trial. *Surg Endosc* Surg Endosc; 2019; **33**: 870–8

46. Yilmaz G, Kiyak H, Akca A, Salihoglu Z. Low-pressure pulmonary recruitment maneuver: Equal to or worse than moderate-pressure pulmonary recruitment maneuver in preventing postlaparoscopic shoulder pain? A randomized controlled trial of 72 patients. *Wideochirurgia I Inne Tech Maloinwazyjne* [Internet] Wideochir Inne Tech Maloinwazyjne; 2020; **15**: 519–25 Available from: 7197

47. Tsai HW, Wang PH, Yen MS, Chao KC, Hsu TF, Chen YJ. Prevention of postlaparoscopic shoulder and upper abdominal pain: A randomized controlled trial. *Obstet Gynecol* Obstet Gynecol; 2013; **121**: 526–31

48. Bundgaard-Nielsen M, Jans Ø, Müller RG, et al. Does goal-directed fluid therapy affect postoperative orthostatic intolerance? A randomized trial. *Anesthesiology* Anesthesiology; 2013; **119**: 813–23

49. Ramsingh DS, Sanghvi C, Gamboa J, Cannesson M, Applegate RL. Outcome impact of goal directed fluid therapy during high risk abdominal surgery in low to moderate risk patients: A randomized controlled trial. *J Clin Monit Comput* J Clin Monit Comput; 2013; **27**: 249–57

50. Liang M, Li Y, Lin L, Lin X, Wu X, Gao Y, Cai H, Zeng K LC. Effect of goal-directed fluid therapy on the prognosis of elderly patients with hypertension receiving plasmakinetic energy transurethral resection of prostat. *Int J Clin Exp Med* 2017; **10**: 1290–6

51. Liu F, Lv J, Zhang W, Liu Z, Dong L, Wang Y. Randomized controlled trial of regional tissue oxygenation following goal-directed fluid therapy during laparoscopic colorectal surgery. *Int J Clin Exp Pathol* e-Century Publishing Corporation; 2019; **12**: 4390–9

52. Luo J, Xue J, Liu J, Liu B, Liu L, Chen G. Goal-directed fluid restriction during brain surgery: a prospective randomized controlled trial. *Ann Intensive Care* [Internet] Springer; 2017 [cited 2021 Oct 25]; **7**: 16 Available from: http://www.ncbi.nlm.nih.gov/pubmed/28211020

53. Noblett SE, Snowden CP, Shenton BK, Horgan AF. Randomized clinical trial assessing the effect of Doppler-optimized fluid management on outcome after elective colorectal resection. *Br J Surg* Br J Surg; 2006; **93**: 1069–76

54. Zhang N, Liang M, Zhang D dan, et al. Effect of goal-directed fluid therapy on early cognitive function in elderly patients with spinal stenosis: A Case-Control Study. *Int J Surg* Int J Surg; 2018; **54**: 201–5

55. Zheng H, Guo H, Ye JR, Chen L, Ma HP. Goal-directed fluid therapy in gastrointestinal surgery in older coronary heart disease patients: Randomized trial. *World J Surg* World J Surg; 2013; **37**: 2820–9

56. Peng K, Li J, Cheng H, Ji FH. Goal-directed fluid therapy based on stroke volume variations improves fluid management and gastrointestinal perfusion in patients undergoing major orthopedic surgery. *Med Princ Pract* Med Princ Pract; 2014; **23**: 413–20

57. Gan TJ, Soppitt A, Maroof M, et al. Goal-directed intraoperative fluid administration reduces length of hospital stay after major surgery. *Anesthesiology* Anesthesiology; 2002; **97**: 820–6

58. Phan TD, D’Souza B, Rattray MJ, Johnston MJ, Cowie BS. A randomised controlled trial of fluid restriction compared to oesophageal Doppler-guided goal-directed fluid therapy in elective major colorectal surgery within an Enhanced Recovery After Surgery program. *Anaesth Intensive Care* Anaesth Intensive Care; 2014; **42**: 752–60

59. Weinberg L, Ianno D, Churilov L, et al. Restrictive intraoperative fluid optimisation algorithm improves outcomes in patients undergoing pancreaticoduodenectomy: A prospective multicentre randomized controlled trial. *PLoS One* PLoS One; 2017; **12**: e0183313

60. Weinberg L, Ianno D, Churilov L, et al. Goal directed fluid therapy for major liver resection: A multicentre randomized controlled trial. *Ann Med Surg* Ann Med Surg (Lond); 2019; **45**: 45–53

61. Zhang J, Qiao H, He Z, Wang Y, Che X, Liang W. Intraoperative fluid management in open gastrointestinal surgery: Goal-directed versus restrictive. *Clinics* Clinics (Sao Paulo); 2012; **67**: 1149–55

62. Mühlbacher J, Luf F, Zotti O, Herkner H, Fleischmann E, Kabon B. Effect of Intraoperative Goal-Directed Fluid Management on Tissue Oxygen Tension in Obese Patients: a Randomized Controlled Trial. *Obes Surg* Springer; 2021; **31**: 1129–38

63. Pillai P, McEleavy I, Gaughan M, et al. A double-blind randomized controlled clinical trial to assess the effect of doppler optimized intraoperative fluid management on outcome following radical cystectomy. *J Urol* J Urol; 2011; **186**: 2201–6

64. Delfino AE, De La Fuente N, Echevarría GC, Altermatt FR, Cortinez LI. Effect of acute arterial hypertension on morphine requirements and postsurgical pain. *J Clin Anesth* J Clin Anesth; 2015; **27**: 226–32

65. Xu XM, Hu XW, Wu Y, et al. Effects of different BP management strategies on postoperative delirium in elderly patients undergoing hip replacement: A single center randomized controlled trial. *J Clin Anesth* J Clin Anesth; 2020; **62**: 109730

66. Kim YS, Won YJ, Lee DK, et al. Lung ultrasound score-based perioperative assessment of pressure-controlled ventilationvolume guaranteed or volume-controlled ventilation in geriatrics: A prospective randomized controlled trial. *Clin Interv Aging* Dove Press; 2019; **14**: 1319–29

67. Le Guen M, Paternot A, Declerck A, et al. Impact of the modality of mechanical ventilation on bleeding during pituitary surgery: A single blinded randomized trial. *Med (United States)* Wolters Kluwer Health; 2019; **98**: e17254
